# REM sleep stabilizes hypothalamic representation of feeding behavior

**DOI:** 10.1073/pnas.1921909117

**Published:** 2020-07-30

**Authors:** Lukas T. Oesch, Mary Gazea, Thomas C. Gent, Mojtaba Bandarabadi, Carolina Gutierrez Herrera, Antoine R. Adamantidis

**Affiliations:** ^a^Zentrum für Experimentelle Neurologie, Department of Neurology, Inselspital University Hospital Bern, CH-3010 Bern, Switzerland;; ^b^Department of Biomedical Research, University of Bern, CH-3010 Bern, Switzerland

**Keywords:** lateral hypothalamus, REM sleep, feeding, calcium imaging, optogenetics

## Abstract

During rapid eye movement (REM) sleep, behavioral unresponsiveness contrasts strongly with intense brain-wide neural network dynamics. Yet, the physiological functions of this cellular activation remain unclear. Using in vivo calcium imaging in freely behaving mice, we found that inhibitory neurons in the lateral hypothalamus (LH^vgat^) show unique activity patterns during feeding that are reactivated during REM, but not non-REM, sleep. REM sleep-specific optogenetic silencing of LH^vgat^ cells induced a reorganization of these activity patterns during subsequent feeding behaviors accompanied by decreased food intake. Our findings provide evidence for a role for REM sleep in the maintenance of cellular representations of feeding behavior.

## Significance

The lateral hypothalamus encompasses neural circuits that are highly active during feeding behavior and rapid eye movement sleep. Yet, it remains unclear whether these mutually exclusive behaviors share common neural populations. Here, we recorded and perturbed the activity of inhibitory neurons of the lateral hypothalamus (LH) across feeding behavior and sleep in freely behaving mice. We found that feeding is reliably encoded by specific patterns of neuron activity and that these patterns are reactivated during rapid eye movement sleep. Disrupting the activity of these inhibitory neurons specifically during rapid eye movement sleep decreased subsequent feeding behavior. These results suggest that rapid eye movement sleep stabilizes the hypothalamic representation of feeding behavior and modulates future food intake.

Rapid eye movement (REM) sleep (or paradoxical sleep) is the most puzzling brain state in vertebrates. It is associated with sensory-motor development and learning (1, 2), memory consolidation (3–5), and dreaming (6). Neural substrates essential for the onset and maintenance of REM sleep have been identified in the ventrolateral peri-aquaeductal gray (7), sublaterodorsal nucleus of the brainstem (8) and in the lateral hypothalamus (9, 10), while the theta rhythm dominating rodent REM sleep electroencephalogram (EEG) largely originates from the septo-hippocampal structure (4). Other hypothalamic, thalamic, and cortical neuron populations also show high activity selectively during REM sleep in rodents (11–15) and humans (16); however, their functions remain unclear.

Among LH neuronal population, vesicular GABA and glycine transporter-expressing neurons (LH^vgat^) show high activity during wakefulness or spontaneous REM sleep (11) and during experimentally induced REM sleep hypersomnia (11, 17). While populations of LH^vgat^ neurons control wakefulness through specific synaptic circuits (18–20), it is still unclear whether LH^vgat^ neuron activity is implicated in REM sleep regulation (21).

Neuronal activity in the LH has been linked to feeding behavior, including motivation (22) and food consumption (23–25). Recent studies have highlighted a critical role for LH^vgat^ neurons in food seeking (26), food intake (27–31), compulsive behavior (28) or hunting (32), and the induction of conditioned place preference in the absence of rewards (27, 33). LH^vgat^ neuron projections to the ventral tegmental area (30, 33), the paraventricular hypothalamus (31), the ventrolateral peri-aquaeductal gray matter (32), and the dorsal pons (34) were shown to modulate feeding and motivation in mice. Taken together, these findings suggest that LH^vgat^ neurons provide a link between sleep and the motivational, or consummatory, aspects of feeding behavior.

Here, we studied the activity of LH^vgat^ neurons as a model to understand the contribution of REM sleep to hypothalamic control of food intake. Using in vivo calcium imaging and optogenetic interventions in freely behaving mice, we recorded the activity of LH^vgat^ neuron populations across feeding and sleeping behaviors and tested the role of REM sleep in the encoding of neuronal representation of feeding behavior.

## Results

### Feeding Is Reliably Encoded by a Stable Map of LH^vgat^ Neuron Activity.

**Fig. 1.**
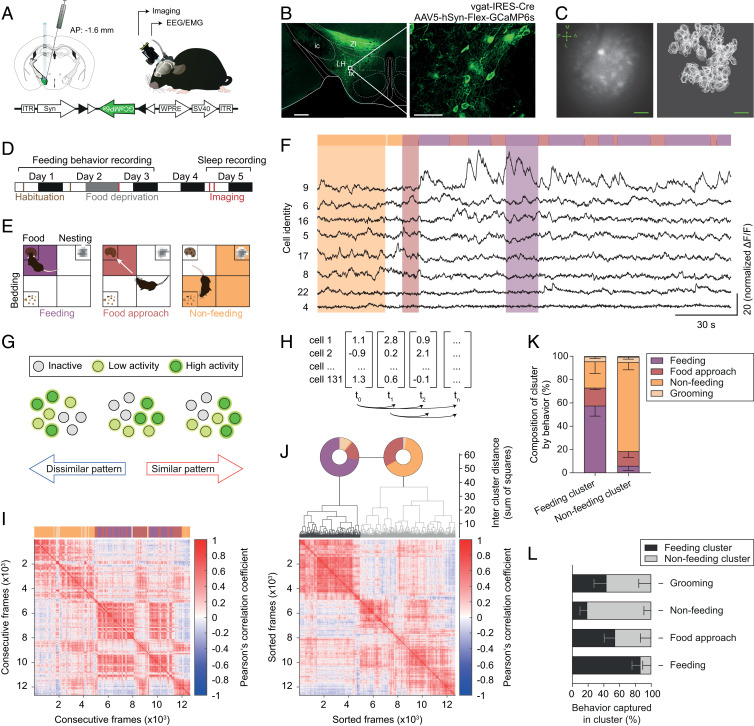
Feeding is reliably encoded by LH^vgat^ neurons. (*A*) Schematic (*Left*) showing LH injection of Cre-dependent AAV in vgat-IRES-Cre mice. Following cre-dependent local virus transfection, the GCaMP6s cassette is flipped in LH^vgat^ allowing transcription and long-term expression in the LH (*Lower*). Illustration of the chronic GRIN lens implantation and imaging using a miniature fluorescence microscope in freely moving mice (*Right*). (*B*) Photomicrograph of cell-specific expression of GCaMP6s in the LH from vgat-IRES-Cre mice 4 wk after virus injection (*Left*). *Right* shows enlargement of the white box highlighted in *Left*. fx, fornix; ic, internal capsule; LH, lateral hypothalamus; ZI, zona incerta. (*C*) Representative field of view from imaging of LH^vgat^ neurons with the miniature microscope (*Left*). Bright cellular structures and dark blood vessels are readily visible. Arrows indicate the body axes (A-P, anterior-posterior; M-L, medial-lateral). Cells were identified using the CNMF-E algorithm (*Right*). Note that the single cell activity was longitudinally recorded across multiple experimental sessions. (Scale bars, *B, Left*, 500 µm; *B, Right*, 50 µm; *C*, 100 µm.) (*D*) Experimental timeline. White and dark boxes represent light and dark phase, respectively. (*E*) An open field arena was divided into four quadrants, which contained either food, bedding, or nesting material or were left empty. Animals were video-tracked and feeding (purple, *Left*), food-approach (red, *Center*), and nonfeeding (orange, *Right*) behaviors were visually scored. (*F*) Representative recording of calcium transients from GCaMP6-expressing LH^vgat^ cells across feeding behavior (color-coded, *Upper*) acquired at 10 frames per second. Eight single-cell recordings are shown (black, *Lower*). (*G*) Schematic illustration of the concept of neural activity-pattern similarity. Note that the number of neurons (circle) with high (green), intermediate (yellow), or no activity (gray) is the same for all of the three frames displayed, while their activity patterns show high (*Right*) or low (*Left*) similarity. (*H*) Matrix of activity-pattern similarity obtained from cross-correlation of the population vectors at all different time points (i.e., imaging frames) with each other. (*I*) Representative similarity matrix for a single animal across free-feeding episodes (color-coded bar, *Upper*). Note the presence of similar activity patterns across consecutive feeding bouts. (*J*) Hierarchical clustering of data shown in *I*. The activity patterns grouped into “feeding” (black) and “nonfeeding” (gray) clusters as shown on the dendrogram. The pie charts (*Upper*) indicate the behavior that was observed when the respective activity patterns occurred. The sorted similarity matrix is shown at the bottom. The clustering is significant, permutation test, *P* < 0.001. (*K*) Mean percentage − SEM of LH^vgat^ neuron activity patterns in the cluster associated with a specific behavior. Note the high specificity of the “feeding” and the “nonfeeding” clusters. (*n* = 5 animals). (*L*) Mean percentage − SEM of frames in the cluster corresponding to specific behavior out of total frames for this behavior (*n* = 5 animals). Note the sensitivity of the respective clusters for the different behaviors.

We first recorded the activity profiles of LH^vgat^ neurons using in vivo calcium imaging from food-deprived, freely moving mice during exploratory behaviors and feeding. To achieve this, we genetically targeted the expression of the calcium indicator GCaMP6s to LH^vgat^ neurons by stereotactically injecting an adeno-associated virus (AAV5) carrying a Cre-dependent expression cassette of GCaMP6s (Syn-Flex-GCaMP6s) into the LH of vgat-IRES-Cre mice (Fig. 1 *A*, *Lower Left*). We observed long-lasting expression of GCaMP6s in the LH area (up to 9 wk; Fig. 1*B*). Note that there was no colocalization of LH^vgat^ with melanin concentrating hormone (MCH)-positive neurons (*SI Appendix*, Fig. S1*A*), another REM sleep-active neuron population in the LH (35, 36). Transduced animals were chronically implanted with both EEG/electromyogram (EMG) electrodes and a gradient refractive index (GRIN) lens for simultaneous sleep–wake recording and optical imaging (up to 8 d) with a miniature fluorescence microscope (37), respectively (Fig. 1 *A–C*, *Right* and *SI Appendix*, Fig. S1*B*). We longitudinally recorded the calcium transients from GCaMP6s-expressing LH^vgat^ neurons, concomitant to EEG/EMG signals and behavioral video-tracking during a free-feeding task that did not involve instrumental learning or behavioral conditioning (Fig. 1 *D* and *F* and Movie S1).

We first investigated whether LH^vgat^ neuron populations reliably encode feeding (i.e., consummatory aspect), food approach (i.e., appetitive aspect), or nonfeeding behaviors using a cross-correlation analysis. Population vectors were computed from the fluorescence intensity values of single LH^vgat^ neurons for each recorded frame of an imaging session and correlated with each other (Fig. 1*H*). Each of these vectors represents the activity pattern of LH^vgat^ neuron population for a single frame, and the correlation indicates the similarity between two frames (Fig. 1*G*). Hereafter, we will use the term “map” to describe the average of LH^vgat^ neuron activity patterns for all frames recorded during feeding, food approach, or nonfeeding behaviors. This analysis revealed that LH^vgat^ neuron activity patterns during feeding were highly similar and that they were distinct from those observed during nonfeeding behaviors (Fig. 1*I*). The robust dichotomy between feeding and nonfeeding activity patterns was further captured by unsupervised hierarchical clustering with high sensitivity and specificity (Fig. 1 *J*–*L* and *SI Appendix*, Fig. S3 *A*–*F*).

**Fig. 2.**
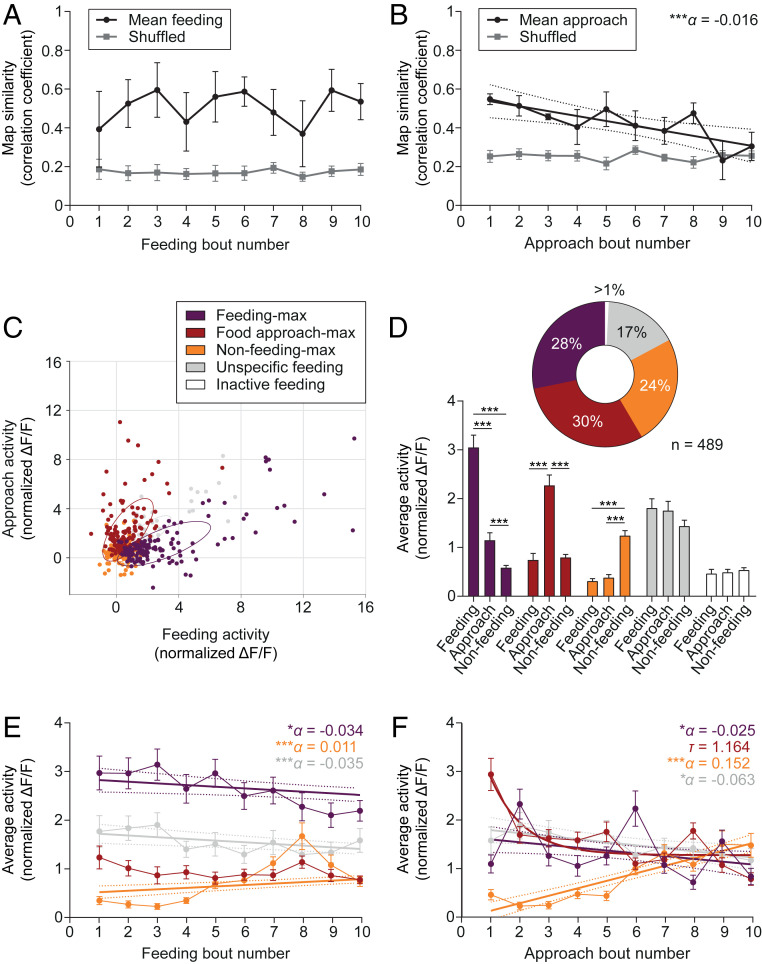
Subpopulations of LH^vgat^ neurons simultaneously carry information about current and prior feeding. (*A* and *B*) Mean ± SEM population vector similarity over consecutive feeding (*A*) or food approach (*B*) bouts during a recording session (black) as compared to 1,000 randomly drawn samples (Shuffled, gray). Similarity was expressed as correlation with the mean feeding or approach vector. The line in *B* represents a significant linear regression ± 95% CI with slope α (*n* = 5 animals). ***P < 0.001. (*C*) Scatter plot showing the average cell activity (normalized Δ*F*/*F*) of each LH^vgat^ cell during feeding and food approach behavior. Neurons were classified according to their activity profiles during different behaviors (*Methods Summary*). Color coding indicates functional clusters. Ellipses represent the mean-centered covariance of the clusters for feeding and food approach behaviors. Note that the graph was projected to the two axes of largest variance. (*D*) Mean + SEM cell activity (normalized Δ*F*/*F*) of GCaMP6s-expressing LH^vgat^ neurons within the different clusters. The pie chart summarizes the classification (*n* = 489 cells from 5 animals, *Upper*). Two-way RM ANOVA, *F*_Cluster(4,__484)_ = 11.70, *F*_Cluster__x__Behavior(8,__968)_ = 13.76, with Tukey’s post hoc test, ***P < 0.001. (*E* and *F*) Mean ± SEM cell activity (normalized Δ*F*/*F*) of LH^vgat^ neurons over consecutive feeding *E* or food approach *F* bouts by functional cluster (*n* = 489 cells from 5 animals). Straight lines indicate significant linear regressions ± 95% confidence interval with slope α for different clusters. Note that the activity of food approach-max neurons in *F* shows a rapid exponential decay with half-life τ. *P < 0.05, ***P < 0.001.

To assess the stability of this representation, we computed an index of similarity by correlating the activity patterns of single frames occurring during individual feeding and food approach bouts to the corresponding map (i.e., averaged activity patterns observed during the respective behavior). We found that the feeding representation was stable over successive feeding bouts (Fig. 2*A*) and independent of a change of overall LH^vgat^ neuron population activity (*SI Appendix*, Fig. S3 *G* and *H*), whereas the one for food approach decreased linearly (Fig. 2*B*). Note that the stability of the representation depends on whether the cells changed their activity in a coordinated or individualized manner (*SI Appendix*, Fig. S4*A*).

We then classified single LH^vgat^ cells based on their activity profiles during food approach and feeding to reflect their contribution to the appetitive or consummatory aspects of feeding behavior, respectively (Fig. 2*C*). We found that subsets of LH^vgat^ neurons were maximally active during food intake (feeding-max, 28%), food approach (food approach-max, 30%), or when animals were outside the food quadrant and not eating (nonfeeding-max, 24%, Fig. 2 *C* and *D*), hereafter referred to as nonoverlapping “clusters.” Some neurons did not show any preferential activity during the scored behaviors (unspecific feeding, 17%, Fig. 2 *C* and *D*). The specificity of this functional classification was further confirmed by within-group post hoc statistical analysis of mean activity level (Fig. 2*D*) and affinity propagation clustering (38), an unsupervised clustering method (*SI Appendix*, Fig. S5 *A*–*C* and *G*–*I*). Interestingly, the activity of feeding-max and unspecific cells linearly decreased over successive feeding bouts, while the activity of nonfeeding-max neurons increased (Fig. 2*E*). Further, whereas the activity of nonfeeding-max neurons increased across food approaches, the activity of the other clusters decreased (Fig. 2*F*). These activity changes between the onset and the end of the recording session led to a decreased specificity of the functional clustering (*SI Appendix*, Fig. S4*B*) and a redistribution of LH^vgat^ neuron clusters (*SI Appendix*, Fig. S4 *C* and *D*).

### Reactivation of Feeding-Related LH^vgat^ Neuron Activity-Patterns during REM Sleep.

**Fig. 3.**
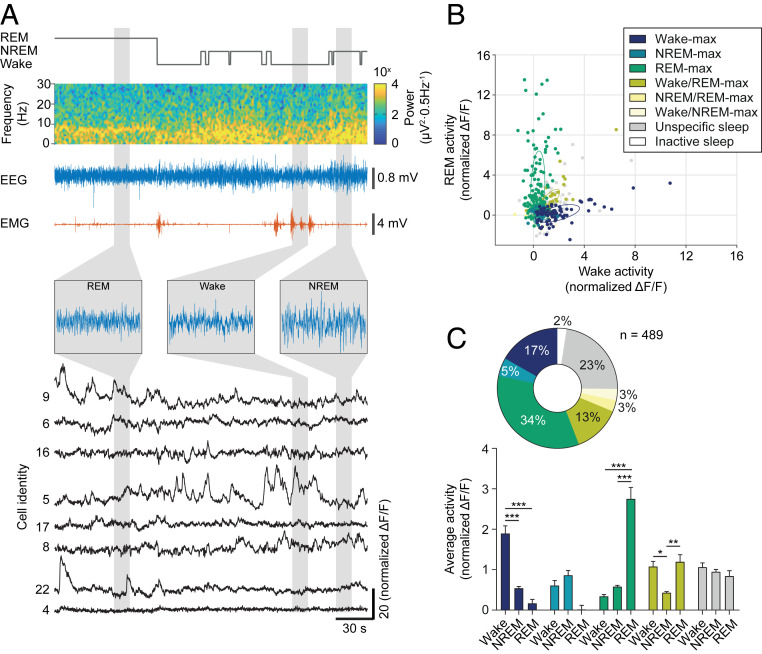
LH^vgat^ neurons show high activity during wakefulness and REM sleep. (*A*) Representative recording of EEG, EMG, and calcium transients from GCaMP6s-expressing LH^vgat^ neurons across sleep–wake states in freely behaving mice. Hypnogram (gray), EEG spectrogram (colormap), EEG (blue), and EMG (red) traces are shown. *Insets* show extended EEG traces for the different sleep–wake states. Calcium transients for neurons identical to Fig. 1*F* are displayed (black). (*B*) Scatter plot shows the average activity (normalized Δ*F*/*F*) of each LH^vgat^ cell during wakefulness and REM sleep. Neurons were classified according to their activity profiles during different sleep stages (*Methods Summary*). Color coding indicates cluster identity. Ellipses represent the mean-centered covariance of the clusters for wakefulness and REM sleep. Note that the graph was projected to the two axes of largest variance. (*C*) Mean + SEM cell activity (normalized Δ*F*/*F*) of GCaMP6-expressing LH^vgat^ neurons within the different clusters encoding sleep and wake states (*Lower*). The pie chart summarizes the classification of LH^vgat^ neurons (*n* = 489 cells from 5 animals, *Upper*). Two-way RM ANOVA, *F*_Cluster__(4,__440)_ = 3.97, *F*_State(2, 880)_ = 5.02, *F*_Cluster__x__State(8, 880)_ = 37.78, with Tukey’s post hoc test, *P < 0.05, **P < 0.01, ***P < 0.001.

To assess the contribution of LH^vgat^ neurons to either wakefulness or REM sleep, we next performed simultaneous recordings of cellular activity and EEG/EMG signals from spontaneously sleeping animals. We observed that the activity of LH^vgat^ neurons was strongly modulated across sleep–wake states (Fig. 3*A*), with subsets of LH^vgat^ neurons maximally active during REM sleep (REM-max, 34%), wakefulness (wake-max, 17%), both wakefulness and REM sleep (wake/REM-max, 13%) or non-REM (NREM) sleep (NREM-max, 5%), and no selectivity for either of the sleep states or wakefulness (unspecific sleep, 23%, Fig. 3 *B* and *C*). The mean activity of neurons within wake-max, REM-max, and wake/REM-max, but not NREM-max or unspecific, clusters was significantly different across sleep–wake states and post hoc comparisons corroborated the classification of LH^vgat^ neurons across sleep–wake states (Fig. 3*C*). Unsupervised clustering further confirmed this result and changes in the classification thresholds mainly led to a reassignment from the wake/REM-max cluster to the wake-max group without significant changes in the other clusters (*SI Appendix*, Fig. S5 *D*–*I*).

**Fig. 4.**
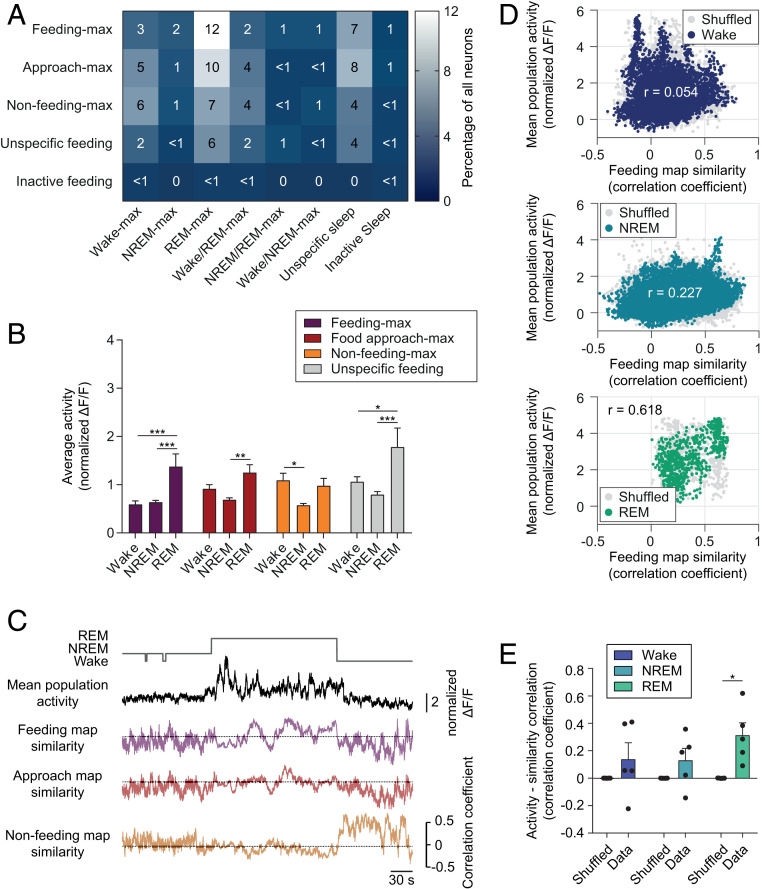
Feeding-like population activity patterns are associated with high activity during REM sleep. (*A*) Coclassification matrix of LH^vgat^ neurons during sleep–wake states and food-directed behaviors. Color coding and numbers represent the percentage of neurons for the corresponding clusters (*n* = 489 cells from five animals). (*B*) Mean + SEM cell activity of the functional clusters for feeding behaviors during the different sleep stages. Note the high activation of feeding-max cells during REM sleep, as compared to wakefulness or NREM sleep. Two-way RM ANOVA, *F*_Cluster(2,__962)_ = 20.90, *F*_Cluster__x__State(6,__962)_ = 2.16, followed by the Tukey’s post hoc test, *P < 0.05, **P < 0.01, ***P < 0.001. (*C*) Representative traces of overall population activity (black) in comparison to map similarity for feeding behaviors (in respective color) over sleep and wake episodes. (*D*) Representative scatter plots show the relationship between feeding similarity and population activity during wakefulness (*Top*), NREM (*Middle*) and REM sleep (*Bottom*). Colored dots represent the true data while for gray dots the population activity was shuffled 1,000 times, *r* indicates the Pearson correlation coefficient for the true data. (*E*) Mean + SEM feeding similarity – activity correlation for true and shuffled data (*n* = 5 animals). Two-way RM ANOVA, *F*_Shuffle(1,__8)_ = 17.10, followed by the Sidak’s post hoc test, *P < 0.05.

Are LH^vgat^ neuron activity patterns during REM sleep similar to those occurring during awake feeding? To first test whether LH circuits contributing to awake feeding behaviors and sleep stages share common cellular substrates, we computed a coclassification matrix of the different LH^vgat^ neuron clusters associated with the feeding task and sleep–wake states (Fig. 4*A*). This revealed a large overlap between the different feeding and sleep clusters (Fig. 4*A* and *SI Appendix*, Fig. S6*A*). We found a significant modulation of the average activity of the neurons in the different feeding clusters across sleep–wake states. Whereas activity of all clusters was lowest during NREM sleep, it was highest during REM sleep for all functional clusters except the nonfeeding-max group (Fig. 4*B*)

At the population level, we next looked at the activity patterns during the different sleep–wake states. We found that the activity patterns of LH^vgat^ neurons during REM sleep showed high similarity to the awake feeding and food approach maps (Fig. 4*C*). While these patterns remained stable over time during REM sleep, they did not during NREM sleep and wakefulness, possibly due to lower activity and higher susceptibility to background noise (Fig. 4*C*). The similarity of the activity pattern to the feeding map was positively correlated with overall cell activity levels during REM sleep, but not NREM sleep or wakefulness, for all of the recorded animals and it was significantly higher than chance (Fig. 4 *D* and *E*). Patterns similar to food approach or nonfeeding maps were not found to be correlated with high population activity for any of the sleep–wake states (*SI Appendix*, Fig. S6 *B* and *C*).

### Optogenetic Silencing of LH^vgat^ Neurons during REM Sleep, but Not Wakefulness, Decreases Food Intake.

**Fig. 5.**
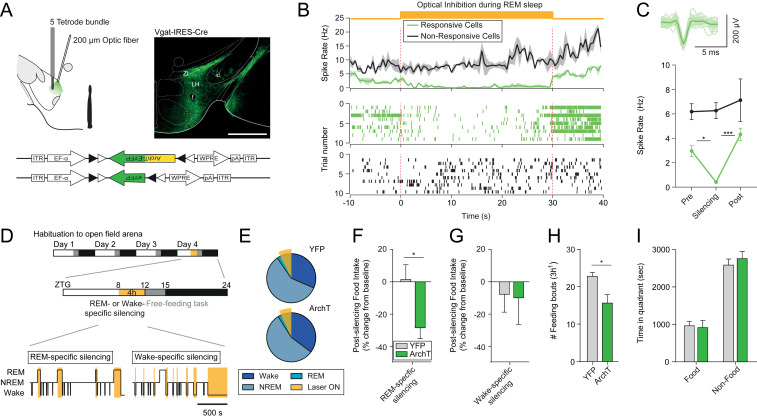
Optogenetic silencing of LH^vgat^ neurons during REM sleep, but not wakefulness, decreases food intake. (*A*) Schematic of optogenetic targeting and tetrode recordings from freely moving mice (*Left*). Photomicrograph shows cell-specific expression of ArchT in the LH of vgat-IRES-Cre mice (*Right*). LH^vgat^ neurons were transfected to either express ArchT-eYFP (test group) or eYFP only (control group, *Lower*). fx, fornix; ic, internal capsule; LH, lateral hypothalamus; ZI, zona incerta. (Scale bar, 500 μm.) (*B*) Average spike rate ± SEM of light-responsive and nonresponsive single units from the LH (*Top*). Continuous light illumination (532 nm, orange shading) was delivered for 30 s during REM sleep. Raster plots from a representative light-responsive (*Middle*) and a nonresponsive (*Bottom*) unit are shown across the illumination period. (*C*) Average waveform of a responsive unit (*Upper*). The mean spike rate of the light-responsive (*n* = 8 cells) and nonresponsive (*n* = 10 cells) units before, during, and after the silencing is shown (*Lower*, *n* = 4 animals). Two-way RM ANOVA, *F*_Responsive(1,__16)_ = 12.69, *F*_Stim(2,__32)_ = 7.24, *F*_Responsive__x__Stim(2,__32)_ = 3.49 with Tukey’s post hoc test, *P < 0.05, ***P < 0.001. (*D*) Timeline of optogenetic silencing experiment (*Top*). Mice were habituated to the open field arena for 3 d prior to testing. Online optogenetic silencing *(Middle)* was conducted between zeitgeber (ZTG) 8 and 12, and food-directed behaviors were quantified in a free-feeding task over the next 3 h (ZTG 12–15). Continuous optical stimulation (orange shading) was delivered selectively during REM sleep (*Bottom Left*) or wake (*Bottom Right*) episodes to silence LH^vgat^ neurons in a state-specific manner. (*E*) Average percentage of vigilance states during the REM-specific optogenetic silencing experiment for YFP control (*n* = 8 animals, *Upper*) and ArchT (*n* = 8 animals, *Lower*) mice. The orange shading indicates total optogenetic stimulation time during REM sleep. Two-way ANOVA, *P* > 0.05. (*F*) Average postsilencing food intake change ± SEM after REM sleep-specific silencing in YFP control (gray, *n* = 8 animals) and ArchT (green, *n* = 8 animals) mice. Student’s unpaired *t* test, *t*_*14*_ = 2.68, *P < 0.05. (*G*) Average postsilencing food intake change − SEM after wake-specific silencing in YFP control (gray, *n* = 5 animals) and ArchT (green, *n* = 7 animals) mice. Student’s unpaired *t* test, *P* > 0.05. (*H*) Average feeding frequency + SEM over the free-feeding task following optogenetic silencing during REM sleep in YFP control (gray, *n* = 6 animals) and ArchT (green, *n* = 5 animals) mice. Student’s unpaired *t* test, *t*_*9*_ = 3.05, *P < 0.05. (*I*) Average time + SEM spent in food and nonfood quadrants of the open field arena after REM sleep-specific silencing for YFP control (gray, *n* = 6 animals) and ArchT (green, *n* = 5 animals) mice. Two-way ANOVA, *F*_Zone(1,__8)_ = 2.60, with Sidak’s post hoc test, *P* > 0.05.

We then tested whether activation of LH^vgat^ neurons during REM sleep contributes to the maintenance of appropriate feeding behavior during wakefulness. To achieve this, we targeted the expression of the light-sensitive proton pump ArchT3.0 to LH^vgat^ neurons by stereotactically injecting an adeno-associated virus (AAV2) carrying a Cre-dependent expression cassette of ArchT (DIO-EF1a-ArchT3.0-eYFP, silencing) or YFP (DIO-EF1a-eYFP, control) into the LH of vgat-IRES-Cre mice (Fig. 5 *A*, *Lower Right*). We tested for the biological function of the opsin by recording the electrophysiological response to optogenetic silencing in mice that were chronically implanted with tetrodes inside a microdrive and an optical fiber in the LH (Fig. 5 *A*, *Left*). Of 18 recorded neurons, 8 showed a significant decrease in spike rate upon continuous illumination delivered selectively during REM sleep episodes (light-responsive, 44%) and 10 did not change their spike rates in response to the light delivery (light-nonresponsive, 56%, Fig. 5 *B* and *C* and *SI Appendix*, Fig. S7 *B* and *C*). Consistent with our imaging results (Fig. 3), we observed that light-responsive putative LH^vgat^ neurons increased their spiking activity at the onset of REM sleep and remained active until the transitions to wakefulness (*SI Appendix*, Fig. S7 *C*–*E*). An additional cohort of mice were implanted with cortical EEG electrodes, EMG wires, and bilateral optical fibers above the LH. These animals were fed ad libitum and habituated to the free-feeding task for three consecutive days. On the fourth day, LH^vgat^ neurons were optogenetically silenced selectively during REM sleep episodes prior to the free-feeding paradigm in both ArchT3.0- and YFP-transduced animals (Fig. 5 *D* and *E*). Interestingly, we found that silencing of LH^vgat^ neurons selectively during REM sleep significantly reduced average food intake during subsequent wakefulness in ArchT-transduced, as compared to control animals (YFP control vs. ArchT: +1.49 ± 9.09 vs. −29.06 ± 6.87%, Fig. 5*F*). Importantly, silencing during wakefulness in a separate set of animals had no effect on food intake (YFP control vs. ArchT: −13.10 ± 12.44 vs. −14.19 ± 17.86%, Fig. 5*G*), suggesting that this behavioral response is specific to REM sleep manipulation of LH^vgat^ neuron activity. Analysis of the behavioral sequence showed that the reduction of food consumption was due to a significant decrease of the initiation of food intake events (YFP control vs. ArchT: 23.00 ± 1.03 vs. 15.80 ± 2.29, Fig. 5*H*) rather than time spent exploring the food quadrant (YFP control vs. ArchT: 946.04 ± 119.72 s vs. 898.76 ± 187.91 s, Fig. 5*I*). The baseline values of food intake were similar between YFP control and the ArchT-transduced mice in both the REM- and the wake-silencing cohorts. Furthermore, neither REM-, nor wake-selective silencing altered the sleep–wake cycle parameters during the optogenetic procedure or the following free-feeding experiments (Fig. 5*E* and *SI Appendix*, Figs. S8 and S9, respectively).

### REM Sleep Stabilizes the Feeding Map.

**Fig. 6.**
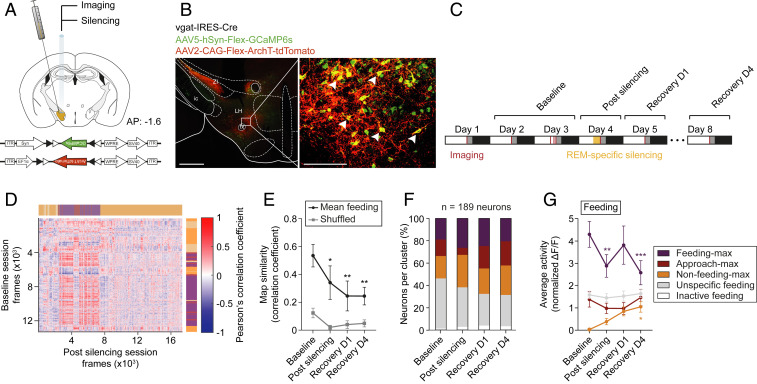
Optogenetic silencing of LH^vgat^ neurons during REM sleep induces long-term changes in the feeding map. (*A*) Schematic of genetic cotargeting of GCaMP6s and ArchT-tdTomato to the LH of vgat-IRES-Cre mice for calcium imaging and optogenetic silencing, respectively. Animals were implanted with a GRIN lens for silencing and recording. The GCaMP6s and ArchT-tdTomato expression cassettes are shown on the bottom. (*B*) Photomicrograph of long-term cell-specific expression of GCaMP6s (green) and ArchT-tdTomato (red) in the LH from vgat-IRES-Cre mice (*Left*). *Right* shows enlargement of the white box highlighted in *Left*; the arrows show GCaMP6s/ArchT-tdTomato double-expressing LH^vgat^ neurons. fx, fornix; ic, internal capsule; LH, lateral hypothalamus; ZI, zona incerta. (Scale bars: *Left*, 500 µm; *Right*, 100 µm.) (*C*) Timeline of optogenetic silencing and calcium recording, concomitant to EEG/EMG measurements for up to eight consecutive days. (*D*) Representative REM sleep population vector correlation matrix before (rows, vertical) and immediately after REM sleep-specific optogenetic silencing (columns, horizontal). Video-tracked and scored behaviors are indicated using color coding. Note that the matrix is not square because the sessions contain a different number of frames. (*E*) True average population vector similarity (black) and shuffled (gray) ± SEM between the feeding vectors across experimental timeline (*n* = 5 animals). Two-way RM ANOVA, *F*_Session(3,__12)_ = 4.23, *F*_Shuffle(1,__4)_ = 10.38 with Dunnett’s post hoc test against baseline, *P < 0.05, **P < 0.01. (*F*) Bar graph shows the classification of imaged neurons across experimental timeline (*n* = 5 animals). (*G*) Average activity ± SEM of the different functional clusters across the experimental timeline for feeding behavior. Two-way RM ANOVA, *F*_Cluster(3,__183)_ = 20.4, *F*_Cluster__x__Session(9,__549)_ = 3.28 with Dunnett’s post hoc test against baseline, *P < 0.05, **P < 0.01, ***P < 0.001.

To investigate whether REM sleep provides a window for activity tuning of neuronal network dynamics, we targeted the expression of GCaMP6s and ArchT to the right LH by stereotactic coinfusion of AAV5-Syn-Flex-GCaMP6s and AAV2-CAG-Flex-ArchT-tdTomato in vgat-IRES-Cre mice (Fig. 6*A* and *Methods Summary*). ∼74% of GCaMP6s-expressing LH^vgat^ neurons coexpressed ArchT-tdTomato (Fig. 6*B* and *SI Appendix*, Fig. S10 *A* and *B*). Mice were then instrumented with a GRIN lens for consecutive REM sleep-specific optogenetic silencing followed by imaging concomitant to EEG/EMG recordings during the free-feeding task (Fig. 6*C* and *SI Appendix*, Fig. S11*A*). We found that optogenetic silencing induced a significant change of the feeding map in comparison to their baseline representation. The change persisted for at least 4 d (Fig. 6 *D* and *E* and *SI Appendix*, Fig. S13 *A*–*C*). This change was not due to daily variation of the feeding map (*SI Appendix*, Fig. S12*A*) or changes in overall neuronal activity (*SI Appendix*, Figs. S12*E* and S13*E*) since both remained stable over days. Note that also the food approach map was significantly changed (*SI Appendix*, Fig. S13*D*), consistent with its variation within recording session (Fig. 2*B*) and across days (*SI Appendix*, Fig. S12*B*).

At the single-cell level, the activity of feeding-max neurons was significantly decreased during postsilencing feeding behavior but not during food approach or nonfeeding behavior (Fig. 6 *F* and *G* and *SI Appendix*, Fig. S13 *G*–*I*). This decrease was not due to changes in the cellular recruitment because the number of LH^vgat^ neurons per cluster remained stable over days, and after optogenetic silencing (Fig. 6*F* and *SI Appendix*, Fig. S12*C*), despite daily variation of the cellular composition of the clusters (*SI Appendix*, Figs. S12*D* and S13*F*). These effects were not due to photobleaching of GCaMP6s fluorescence upon laser light stimulation (*SI Appendix*, Fig. S11 *B*–*I*).

## Discussion

In 1980, Michel Jouvet speculated that REM sleep may decrease the interindividual variability of innate behaviors within a strain. He proposed that neuronal activity driving innate behaviors might be reprogrammed during REM sleep (39). Our findings are in line with this hypothesis and provide a cell-to-behavior framework in support of a role for REM sleep in the stabilization of the neuronal substrate encoding feeding behavior.

At the population level, our results show that feeding behavior is reliably associated with a unique set of highly similar patterns of LH^vgat^ cell activity (here called “map”) that are stable within, and between, behavioral sessions, but distinct from those observed during nonfeeding behavior. Thus, this map captures both active and inactive LH^vgat^ neurons that may ultimately represent an optimal state of LH^vgat^ neurons to control feeding through their downstream synaptic targets. Indeed, LH^vgat^ neurons that modulate circuits implicated in attention (18), arousal (18–20), and reward (30, 33), all of which are essential in the elaboration of a feeding behavior, are strongly active during food consumption. However, other LH^vgat^ neurons implicated in mutually exclusive behaviors are presumably silent, as shown in our study. These distinct patterns of LH^vgat^ neurons activity—ranging from low to high activity—are thought to be essential for the proper encoding of behavior. Consistent with this, distinct LH^vgat^ neuron pathways exert indirect activation and direct inhibition on dopaminergic neurons of ventral tegmental area (33). Therefore, increasing dopaminergic neuron activation could be mediated by increasing the indirect pathways and decreasing the direct ones, leading to a map encompassing both high and low activity LH^vgat^ neurons. In line with this hypothesis, we showed that the map observed during feeding is remarkably stable in time and independent of prior food intake, suggesting that LH^vgat^ neuron modulation of downstream synaptic pathways is preserved over days, motivational and homeostatic states. In contrast, maps observed during food approach behavior are prone to change, possibly reflecting the need for rapid adaptation during food seeking behaviors. Although the food approach representation was not stable in our free-feeding tasks, we cannot rule out that it may become more reliable during an instrumental learning task where animals are trained to repeatedly perform the same behavior (27).

At the single-cell level, we identified clusters among LH^vgat^ neurons with maximal activity during food approach, feeding, or nonfeeding behaviors, consistent with previous reports (27). We showed that these clusters progressively change over successive feeding bouts; while the activity of feeding-max LH^vgat^ neurons progressively decrease across consecutive feeding bouts, the activity of nonfeeding LH^vgat^ neurons increases, suggesting a rapid adaptation of LH^vgat^ neurons during ongoing behavior. Altogether, these findings indicate that the LH^vgat^ population activity map provides a global signal for feeding that is stable over time, while the activity of single LH^vgat^ neurons may reflect a rapid tuning of motivational or homeostatic drives.

Our observation that neuronal activity during REM sleep positively correlates with similarity to the feeding map supports a role for REM sleep in the stabilization of feeding behavior. It suggests a Hebbian mechanism, by which the activity pattern that signals “feeding” during wakefulness is strengthened during REM sleep. Noteworthy, the strengthening of the feeding map via activity tuning of LH^vgat^ neurons may occur sporadically, possibly tied to REM sleep-specific network oscillatory events (26, 39–41). In agreement with this view, silencing of LH^vgat^ neuron activity during REM sleep led to long-lasting changes of the feeding map, resulting in a decrease of feeding bout number and overall food consumption. These changes of the feeding map are reflected by a decrease in the activity of feeding-max neurons concomitant to an increase in the activity of nonfeeding-max during feeding behavior after optogenetic silencing. Yet, it remains to be investigated whether the REM sleep-dependent pattern strengthening described here relies on selective spine turnover of LH^vgat^ cells as shown for layer 5 pyramidal neurons during sleep-dependent consolidation of a motor task (1) or whether it involves other synaptic mechanisms.

This consolidation may differ from the classical hippocampus-dependent memory consolidation that is thought to incorporate novel, but not familiar, experience into mnemonic traces during NREM sleep (42, 43). Indeed, our findings are suggestive of a regular readjustment (e.g., “daily resetting” during REM sleep) of the intrinsic representation of feeding behavior and a constant optimization of the behavioral command. We speculate that a “sleep reset” would represent an ideal mechanism to revert day-to-day adaptative changes of LH^vgat^ neuron activity patterns similar to what have been observed during REM sleep modulation of hippocampal neurons (44) or prefrontal network excitability (45). Whether REM sleep offers a window for offline recalibration of the internal representation of behaviors other than feeding remains to be investigated.

Collectively, these data show that REM sleep activation of LH^vgat^ cells strongly contributes to the maintenance of food intake and feeding initiation during future wakefulness.

## Methods Summary

Animals were treated according to protocols and guidelines approved by the veterinary office of the Canton of Bern, Switzerland (license no. BE 113/13). We recorded male vgat-Cre mice (46) aged between 15 and 36 wk. The animals were kept under a 12/12-h light–dark cycle. Two weeks after the viral transfection the mice were implanted with either a GRIN lens and EEG and EMG electrodes or optical fibers and tetrodes plus EEG and EMG leads. Another 2 wk later the animals were habituated to the miniaturized microscope and the tethers for at least 3 d before recording.

To assess the feeding behaviors, animals were exposed to a square, 29.7 by 29.7 cm, open-field arena. In three corners of the arena either food, the animals’ bedding, or their nesting material were presented. One corner was left empty. Animals were video tracked, and their behavior and position were visually scored. Sleep recordings were performed in the open top home cages (18 × 29 cm) of the mice. Sleep stages were scored offline based on EEG and EMG measurements.

To extract the calcium transients for the individual neurons, the calcium movies from all of the acquired sessions were motion-corrected and aligned to a reference image and finally concatenated. Single neurons and their calcium signals for each mouse were extracted using the CNMF-E toolbox (47) from the entire dataset obtained from the respective animal. The normalized fluorescence (normalized Δ*F*/*F*) was obtained by first subtracting an estimate of the baseline fluorescence from the signal and then dividing it by its noise envelope. Using this approach, the time series captured when neurons are inactive is approximately zero-mean and has unit standard deviation and cellular activation is characterized by transient activity peaks > 2 normalized Δ*F*/*F*.

The similarity between activity patterns was assessed using Pearson’s correlation coefficient between population vectors. To test whether pattern similarity was higher than chance, the signal was shuffled in the time domain (referred to as permutation test), retaining the respective identities of individual neurons. For comparisons of the activity levels between cells and cell groups, we used the average fluorescence and performed ANOVA.

For more details on surgical procedures, behavioral paradigms, and signal acquisition and processing, refer to *SI Appendix*, *Materials and Methods*.

## Supplementary Material

Supplementary File

Supplementary File

## Data Availability

All presented data and analysis scripts, including mat-files and Matlab scripts and functions, are available on Dryad: https://doi.org/10.5061/dryad.kh189323d (48).
