# Supplementary material for: REM sleep stabilizes hypothalamic representation of feeding behavior
Source: Proc Natl Acad Sci U S A. 2020 Jul 30;117(32):19590–8. doi: 10.1073/pnas.1921909117 (PMC7430996; doi:10.1073/pnas.1921909117)
Supplement: Supplementary File [file pnas.1921909117.sapp.pdf]

PNAS
www.pnas.org

Supplementary Information for:

REM sleep stabilizes hypothalamic representation of feeding behavior

Lukas T. Oesch, Mary Gazea, Thomas C. Gent, Mojtaba Bandarabadi, Carolina Gutierrez Herrera and Antoine R. Adamantidis

Corresponding author: **Antoine R. Adamantidis**
Email: **antoine.adamantidis@dbmr.unibe.ch**

This PDF file includes:

SI Materials and Methods
Figures S1 to S13
SI References

Other supplementary materials for this manuscript include the following:

Movie S1

SI Materials and Methods

Animal subjects

We used male Slc32a1$^{tm2(cre)Lowl}$ (vgat-IRES-Cre) mice maintained on a 129/SvJ x C57/B6j background with five generation of backcrossing to the C57/B6j (1). Behavioral experiments were conducted in animals aged between 15 and 36 weeks. Before surgeries the mice were group-housed in ICV cages (Green Line, Techniplast) and provided with a shelter, nest building material, a wood piece, chow food (3432PX, Kliba-Nafag) and water *ad libitum*. After surgery the animals were individually housed in custom, open polyacryl cages (18 x 29 cm) and had access to the same items as stated above except for the shelter. In both conditions the room temperature was maintained at 22 ± 1 °C and humidity allowed to fluctuate between 40-60 %. The rooms were illuminated according to a 12 h light-dark cycle. Depending on the type of the experiment the lights-on time was 8 (for the imaging and optogenetic silencing experiments) or 3 AM (for the optical silencing and imaging experiments). A final number of 5 out of 8 vgat-IRES-Cre mice were included in the imaging experiments with 97.8 ± 66.6 neurons recorded per animal (mean ± standard deviation). For the optogenetic silencing experiments a number of 30 mice were randomly assigned to either the ArchT or the YFP condition to yield a final count of 8 ArchT and 8 YFP mice for the REM-specific silencing and 7 and 5 mice, respectively for the wake-specific silencing with satisfactory expression and fiber optic placement. The experimenters were not blinded to the assigned condition. The photo-bleaching of GCaMP6s fluorescence after laser stimulation was assessed for 2 animals. Finally, 5 out of 19 implanted mice that displayed satisfactory signals and co-expression of GCaMP6s and ArchT-dtTomato completed the recording schedule and were therefore included into the experiments for combined optogenetics and calcium imaging. We recorded 37.8 ± 36.0 (mean ± standard deviation) neurons from these five animals.

All described experimental procedures with animals were approved by the veterinary office of the Canton of Bern, Switzerland (BE 49/17).

Viral targeting

For the *in vivo* imaging and the optogenetic experiments we only included males; for anatomically characterizing GCaMP6s expression and co-localization with MCH we used both male and female mice. 5 – 7 week- old vgat-IRES-Cre mice were anesthetized with 5 % isoflurane for induction and later maintained at 1.5 – 2 %. The mice were placed in a Kopf digital stereotactic frame and their body temperature was kept constant at 37 °C via a feedback-coupled heating device (Panlab/Harvard Apparatus). Eye ointment (Bepanthen Augen- und Nasensalbe, Bayer) was used during the surgery and a subcutaneous bolus injection of meloxicam (2 mg/kg, Boehringer-Ingelheim) was given prior to the surgical procedure. After skin incision, the skull bone was cleaned with 4 % hydrogen peroxide and saline to remove remaining tissue. A small hole was drilled into the skull and the dura was carefully removed. For calcium imaging experiments, we injected 0.6 µl of recombinant AAV5-Syn-DIO-GCaMP6s-WPRE-SV40 (2) into the lateral hypothalamus at the following coordinates from Bregma (3) anterior-posterior (AP): -1.3

mm, medial-lateral (ML): ±1 mm, dorso-ventral (DV): -5.25 mm. The virus was packaged by the Penn Vector Core (University of Pennsylvania, USA) at titers between $3\cdot10^{13}$ to $4\cdot10^{13}$ cfu per ml. For optogenetic silencing of LH^{vgat} neurons, vgat-IRES-Cre mice were randomly assigned to viral injections; one group received AAV2-EF1a-DIO-eArch3.0-eYFP, a silencing opsin (4), and another set of animals received AAV2-EF1a-DIO-eYFP (control, both virus vectors were packaged at the Vector Core of the University of North Carolina at Chapel Hill, titers between $2\cdot10^{12}$ to $4\cdot10^{12}$ cfu per ml). 0.6 µl of each virus was injected bilaterally into the lateral hypothalamus at the following coordinates: AP: -1.4 mm, ML: ±1 mm, DV: -5.4 mm. For combined optogenetic perturbation and calcium imaging we diluted AAV2-CAG-Flex-ArchT-tdTomato-WPRE-SV40 1:25 in the GCaMP6s virus preparation and injected it at the same location as mentioned above for the imaging experiments. The virus suspension was infused with a micro-infusion pump (PHD Ultra, Harvard Apparatus) through a 28 G stainless steel cannula (Plastic One) at a rate of 0.1 µl per minute.

Surgical procedures for imaging and polysomnographic recordings

Two weeks after virus injection, mice were anesthetized and placed in the stereotactic frame as described above. For all experiments including imaging, a 0.5 mm diameter and 8.4 mm length gradient refractive index lens (GLP-0584, Inscopix,) was slowly inserted into the brain to target the following coordinates from Bregma: AP: -1.6 mm, ML: +1 mm (right hemisphere), DV: -4.9 mm. Note that the implantation site is located slightly posterior to the injection site to avoid imaging excess viral debris and more dorsal because the focal plane of the lens on the image side lies roughly 0.25 mm below the lens surface. The lens was cemented to the skull with a three-component dental cement (C&B-Metabond, Parkell Inc.). Four stainless steel screws were inserted into the skull for recording EEG signals (two into both frontal regions, one into parietal left and one into occipital right region) and wire loops were sewed to both neck muscles for EMG recordings. Screws and wires were then covered with a layer of black dental cement (Neocryl Orthodontic Resin, Keystone) and a cement plateau was built around the lens. The top of the GRIN lens was covered with the lid of an Eppendorf tube.

After a minimum of two weeks of recovery the animals were anesthetized again and the optimal imaging plane for the miniature microscope (nVista 2, Inscopix) was determined. Then, a baseplate to attach the miniature microscope was cemented onto the existing cement layer with black dental cement.

Tetrodes were assembled by twisting four strands of insulated 10 µm tungsten wires (CFW0010954, California FineWire) and were attached to a 32-channel electrode interface board with gold pins. Five tetrode bundles were glued into 360 µm diameter silica tubing (TSP-250350, BGB), and the tube was attached to a micro-drive (Nanodrive, Cambirdge NeuroTech). The tetrodes were inserted into the brain of vgat-IRES-Cre mice previously transfected with AAV2-EF1α-DIO-ArchT3.0 at the following coordinates from Bregma: AP: -1.4; ML: +1.0, DV: -4.8 mm. Tetrodes were lowered 100 µm deeper into the brain per day, corresponding to 0.5 screw turns until the tips reached DV: -5.6 mm from Bregma. An optical fiber implant (200 µm, 0.39 NA Core Multimode Optical Fiber, FT200EMT, TECS Clad, Thorlabs, inserted into zirconia ferrules, 1,25 mm OD; 230 µm ID, Precision Fiber product) was implanted with an anterior tilt

above the LH to deliver laser illumination close to the tetrode position (AP: -1.7; ML: +1.0; DV: -5.4, angle: 15°). EEG and EMG electrodes were implanted as described above.

For optogenetic silencing and free-feeding experiments, vgat-IRES-Cre mice were chronically implanted with bilateral optical fiber implants above the lateral hypothalamus (AP: - 1.4; ML: ± 1.0 mm; DV: - 5.0 mm), which were fixated to the skull with C&B-Metabond (Parkell Inc.). In addition, four EEG screws and two EMG electrodes were implanted as described above. The EEG/EMG and optic fiber implant were secured to the skull with dental cement (Paladur, Patterson dental). After surgical procedures mice were allowed to recover for two weeks.

Data acquisition

Before the start of the experiments the animals were habituated to a dummy miniature microscope, the optical patch cords and EEG/EMG cables or the electrode board head stage (RHD2132, Intan Technologies) for at least 3 days or until the animals recovered a normal sleep-wake cycle. Subsequently a 24-h baseline polysomnographic recording was obtained without imaging or optogenetic manipulations. Before the start of an imaging experiment (that could contain multiple sessions) the mice were restrained to remove the microscope dummy and to attach the miniature microscope. After the restraint, animals were allowed to calm down for at least 5 minutes in their home cage before data acquisition. Microscopy data were obtained at 10.01 frames per second at a typical excitation light power of 0.313 mW/mm^2 (20 % of maximal power, nVista 2, Inscopix). EEG and EMG signals were sampled with an AM systems 3500 amplifier at 512 Hz and digitized with a national instruments USB X DAQ device. The animal behavior was filmed using a FireFly MV camera (Point Grey Research) at 15 frames per second. EEG and EMG data, as well as the behavioral video were acquired with the SleepScore software (View Point). After data acquisition, the calcium imaging data were aligned to the polysomnography via recorded time stamps for each frame. Due to the large-scale data streaming the length of an individual imaging session was restricted to approximately 20 minutes. The imaging experiments without optogenetic perturbation took place between zeitgeber time (ZTG) 4-7 the intervention experiments between zeitgeber time 8-15, see below. For each animal, the focus of the miniature microscope remained unchanged and the same field of view was imaged during all the experiments.

Tetrode signals were acquired at 20 kHz with an open-source acquisition software (Open Ephys) via a digitizing head stage (RHD2132) and a multi-channel DAQ board (Open Ephys, Intan Technologies).

Image processing and cell detection

Stacks of calcium imaging data were first preprocessed to fix time-invariant pixels or reconstruct frames lost during the acquisition and then spatially binned 16x (4x each dimension) using Mosaic 1.1.3v (Inscopix). Then the image stacks were corrected for motion artifacts in x-y direction. For this purpose, we selected the most stable calcium movie per animal and computed a mean projection of a timespan within this movie with the least possible detectable movements. This projection was then used as a reference image to motion-correct and align all the imaging data obtained from the same animal. To correct the

images we used a gradient-based registration approach that shows a robust performance even in regimes where brightness fluctuates largely (5). Because of the high fluorescence background in the images we spatially band-pass filtered the data between 2 and 20.5 (already binned) pixels, corresponding to approximately 4 to 42 µm, to compute the shifts. Since some large shifts can be introduced by the plugging and unplugging of the scope we allowed for a static visual pre-alignment of the movies in order to 'initialize' the registration algorithm with a plausible start position. The corrected image stacks for every behavioral paradigm were then concatenated. Joining the image stacks served the purpose to increase the detection sensitivity and specificity and rendered cell tracking and re-finding obsolete.

Putative neurons were first detected by identifying seed pixels with high estimated signal-to-background ratio and by adding neighboring pixels with high temporal correlation with respect to the seed to the shape of the neurons. Then, a linear model was fit that sought to reconstruct the individual frames from the putative cellular sources, a static and a dynamic background and some high frequency noise. After a manual curation and exclusion of artifacts the model was fit again. These procedures have be described previously (6).

To estimate the baseline fluorescence level (F_0) of each trace and mean-center signal windows corresponding to putative cell inactivity, we used a two-step procedure. First, due to the potential bleaching of the GCaMP6s fluorescence within the session we fitted and subtracted an exponential curve from the raw signal of each cell *i* during session *j* and considered this bleaching adjusted version alongside the raw trace (***SI Appendix*, Fig. S2*C***). Second, we approximated the distribution of the observed intensity values from each cell in each session as a mixture of a number of Gaussian distributions via an expectation-maximization procedure (***SI Appendix,* Fig. S2*C***). Among the fitted distributions the mean (μ) of the distribution containing the lowest values was the F_0 for neuron *i* and session *j*. From visual inspection we determined a number of three Gaussians to yield good estimates for F_0 over a broad range of activity regimes, ranging from inactive over sparsely active to highly active neurons (***SI Appendix,* Fig. S2*B***). Similarly, we fitted the Gaussians for the de-trended signals (***SI Appendix,* Fig. S2*C***). If trends are present in the baseline of the signal they will likely increase the variance of the lowest of the Gaussians, that is, our candidate for F_0. Therefore, if the de-trending decreased this variance, we considered the de-trended signal. The F_0 was then subtracted from each trace.

Since the classical ΔF/F tends to be distorted for F_0 values between -1 and 1 (in regimes with high background as often observed in miniscope data) we took another approach to scale the calcium traces and based it on the following assumptions: 1. Due to slow kinetics of the GCaMP6s fluorescence the amplitude of the intensity changes is more bounded than the absolute intensity itself and is thus less affected by the neuron's activity regime. 2. Given 1., the median positive envelope, E_{ij}, of the first order derivative of the signal from each neuron i during session j would be reasonable estimate of the 'noise' in the calcium signal (***SI Appendix,* Fig. S2*D***):

$$E_{ij} = median\left(\left|hilbert\left(\frac{dF_{ij}(t))}{dt}\right)\right|\right)$$

Where $dF_{ij}(t)/dt$ represents the first derivative of the fluorescence time series of neuron i for the respective session j (implemented practically as the fluorescence difference between different frames) and $|hilbert(\,)|$ the real valued part of the Hilbert transform. Dividing each neuron's session time series by its respective scaling factor, E_{ij}, resulted in a normalized fluorescence intensity estimate, referred to as 'normalized ΔF/F' throughout the paper. The final normalized signal closely resembles zero-mean Gaussian noise with a standard deviation of one during stationary, 'noisy', periods and non-stationary epochs of high calcium transients where the signal typically exceeds values of 2 for all neurons (***SI Appendix,*** **Fig. S2*E*** and ***F***).
Unless indicated otherwise all image and calcium signal processing was done in Matlab using custom written scripts.

Single unit isolation and analysis
We first extracted the multi-unit activity by applying and elliptic band-pass filter from 600 to 4000 Hz. The filtering was applied in both, forward and reverse direction to avoid introducing phase shifts in the signal. A spike detection threshold of 7.5x the median filtered signal was set and the remaining multi-unit activity was sorted using the WaveClus toolbox (7) to isolate similar waveforms belonging to putative single units. In brief, a discrete four-level Haar-transform was applied to the signal and the ten most discriminative wavelet coefficients were selected using the Kolmogorov-Smirnov test. The selected coefficients were then clustered using super-paramagnetic clustering, to obtain potential single-units (7). Spikes that were attributed to an isolated single unit were overlaid and carefully inspected. Putative single units with symmetric spike waveforms or low firing rate (< 0.2 Hz) were discarded.
The identified single units were classified as light responsive when there was a complete cessation of activity during green light (532 nm) delivery during REM sleep and were regarded as non-responsive otherwise. We plotted the light-induced changes in spike rate for light responsive versus non-responsive neurons (**Fig. 5*C***) as well as mean spike rate during the different sleep-wake states (***SI Appendix,*** **Fig. S7*C***) and the spiking across transitions into and from REM sleep (***SI Appendix,*** **Fig. S7*D*** and ***E***) in non-stimulated conditions.

Behavioral paradigms
*Free-feeding experiment (**Figure1**, **Figure 2** and **Figure 4**)*
For the feeding imaging experiment the mice were habituated to an open field arena. The arena was a square 29.7 x 29.7 cm box (**Fig. 1*E***). Three corners contained a plastic tray (85 cm^3) filled with either bedding, nesting material or food pellets, one corner remained empty. An empty tray was placed in the corner of the food for the first 5 minutes of the recording and later exchanged with a food-containing one. The behavior of the animal inside the arena was recorded for 20 minutes using a FireFly MV camera (Point Grey Research) at 15 frames per second. The day before the first imaging the animals were habituated to the open field for 20 minutes. One day after the mice were imaged (up to 2 sessions) and

recorded as they moved and ate in the open field. Animals were food-deprived for 16 hours prior to the imaging session (**Fig. 1*E***).

The filmed behavior was scored visually for each second. We quantified the position of the animals in the arena and its food intake. Specifically, we aimed to distinguish feeding from other food interactions, such as smelling, whisking or touching the food.

*Sleep-wake cycle experiment (**Figure 3**)*

2-3 imaging sessions were recorded as the mice were naturally sleeping and waking in their home cage at ZTG time 5-7. No videos were acquired together with these recordings.

*Free-feeding paradigm following optogenetic silencing (**Figure 5** and **Figure 6**)*

For optogenetic silencing experiments, the mice were habituated to the open field arena described above for three consecutive days during the first three hours of the dark period (ZTG 12 to 15). Their 3-h food intake was quantified and their behavior in the arena was recorded on the last day of habituation. On the fourth day, optogenetic silencing of LH^{vgat} neurons was performed specifically during REM sleep or during wakefulness (for bouts longer than ~10 s) for the last four hours of the light period. Afterwards, animals were again placed in the open field arena for three hours, during which their food consumption and behavioral parameters were monitored. Animal behavior was scored using Ethovision XT video tracking software (Noldus) and was corrected manually when animals could not be tracked automatically.

In the experiments with imaging and optogenetic stimulation the animals were imaged at the beginning of the free-feeding task for 20 minutes or until they ate. Their behavior was scored only for the time we imaged the cell activity. Additional imaging sessions were carried out one and four days after the silencing.

Sleep scoring and time-frequency analysis

The acquired EEG and EMG data were scored as described previously (8) (**Fig. 3*A***). Briefly, we defined wakefulness as periods with low-amplitude desynchronized EEG signals and high, tonic EMG activity containing phasic bursts. NREM sleep was characterized by synchronized, high amplitude oscillations in the slow- and delta band and low EMG tone without bursts. REM sleep was scored when the EEG showed pronounced theta oscillations or an increased amount of desynchronized and high-frequency oscillation with almost complete absence of EMG tone except short twitches. The 24 h baseline recordings were scored in epochs of 5 s. The recording sessions were scored at an epoch duration of 1 s. All power spectrum computations were obtained using the *hilbert* function in Matlab.

Optogenetic silencing during polysomnographic recordings

For *in vivo* optogenetic stimulation experiments, a 3-m-long fibre optic patch cord with protective tubing (Thorlabs) was connected to the chronically implanted optical fiber with a zirconia sleeve (Precision Fiber Products). The patch cord was connected to a 532nm DPSS laser (S532005FX, Laserglow Technologies)

via a FC/PC connector (250 um, 30126G2-250, Thorlabs). The laser output was controlled using a Master-9 pulse generator (A.M.P. Instruments, LTD). Optical silencing of LH^{vgat} neurons was achieved by applying continuous illumination at a power output of up to 30 mW/mm^2 from the tip of the optic fibers measured with a power meter (PM100A, Thorlabs). Optical silencing was state-specific based on visual inspection of the animal behavior and online EEG as well as EMG signal parameters over the last four hours of the light period (ZTG 8 to 12, **Fig. 5*D***). For the REM sleep-specific optogenetic silencing experiment, the optical silencing started ~ 5 s after the onset of REM sleep and stopped immediately after REM sleep-to-wake transition. For wake-specific silencing experiment, the optical stimulation started 10 s after wake onset (to exclude micro-arousals) with a 5-s ON/5-s OFF pattern throughout the wakefulness episode or continuously ON for up to 50 s with an approximate cycle of seven minutes. In any case, optical stimulation was stopped when NREM sleep occurred. Note that the same amount of light was delivered for both the REM sleep and wake silencing experiments (REM stimulation *vs* wake: 948.1 ± 66.93 *vs* 1158 ± 232 s, or 726.2 ±114.3 s, for ON/OFF or 50-s continuous illumination, respectively, one-way ANOVA followed by Dunnett's *post-hoc* test, $P > 0.05$ for both conditions). Mice lacking viral expression were excluded from the cohort (n = 2 in total).

Combined imaging and optogenetic silencing

For the experiments that combined silencing and imaging (**Fig. 6** and ***SI Appendix,*** **Fig. S12** and **S13**) animals expressing GCaMP6s and ArchT-tdTomato in LH^{vgat} neurons were implanted with a GRIN lens and a baseplate as described above. For the optogenetic silencing we modified a dummy miniscope such that a fiber-optic patch chord could be inserted into a guide canal drilled through the dummy scope body and secured with a screw (***SI Appendix,*** **Fig. S11*A***). The canal was located centrally, so that the tip of the patch chord was centered on top of the GRIN lens. Before the silencing we exchanged the fluorescence microscope with the modified dummy, inserted the patch chord until it touched the surface of the GRIN lens and secured it in this position with the screw. After our intervention we replaced the dummy with the real microscope to image cell activity. We used a laser output power of 10 mW/mm^2 for these experiments, which was found not to impact on fluorescence intensity levels or the detection of cellular calcium transients (***SI Appendix,*** **Fig. S11*B* - *F***). The protocol was the same as the REM-specific silencing described above otherwise.

All the silencing was performed in the animal's home cage.

Imaging data analysis

Hierarchical clustering of population vectors and pattern similarity

For the population vector analysis we considered the normalized $\Delta F/F$ value of each neuron at a given time point. We did not perform any inference on action potential firing underlying the recorded fluorescence signal (9, 10) because of the combination of two factors: First, vgat-expressing neurons in the LH show a large spectrum of firing rates *in vivo* during sleep (11) and behavior (12). Second, GCaMP6s has a rather slow rise time around 200 ms (2) potentially masking the occurrence of spike

bursts. Hence, spike detection in our experiments would become unreliable when the firing rates get close to, and above, approximately 5 Hz.

We clustered population vectors for the different frames according to their correlation distance (**Fig. 1*I***). First, we generated a distance matrix representing the difference of any two time points as one minus the correlation for these two points using the *pdist* function from Matlab. We then transformed the distance matrix to Euclidean distance using the cosine theorem: $\sqrt{2D_{ij}}$, where D_{ij} is the correlation distance between frame i and j. Then we computed the links using Ward's method with the *linkage* function. We then clustered the values into two separate groups using *cluster* and visualize them with *dendrogram*. For the representation of the sorted correlation matrices we found the order matching the dendrograms using *optimalleaforder* and sorted the data accordingly before re-computing the matrix. Whether the separation into two clusters was significant was tested for by independently shuffling the frame identity for each cell individually 1000 times and re-clustering the data. For all animals the 'height' (the inter cluster distance) at which the dendrogram split into two was larger than any one of the 1000 shuffles, thus $P < 0.001$.

To assess the similarity of activity patterns to a standard representation (i.e. the map) we correlated the instantaneous population vector with the average vector for all observed instances of the respective behavior using *corrcoef* (**Fig. 2*A*** and ***B***, **Fig. 6*E***, and ***SI Appendix,*** **Fig. S12*A*, *B*** and **S13*D***). We also generated a shuffled dataset by randomly drawing the same number of observations from the entire recording session and correlating these to the average vector 1000 times. Note that the shuffled dataset could contain observations during any scored behavior and the true class was not excluded.

Cell classification

To separate cells with distinct activity profiles (**Fig. 1*F*** and **Fig. 2*C*, *D***), we defined the following five categories: Neurons with maximal activity during feeding (feeding-max), during periods where the mice were inside the food quadrant or entered it followed by successful feeding (food approach-max), or whenever the animal was outside the food quadrant and did not eat (Non-feeding-max). Unspecific or inactive cells were identified as such. For the classification we first computed trace of significant calcium events. We set the threshold to 2 normalized ΔF/F (corresponding to approximately 2 noise standard deviations) and assumed a minimal decay time ($\tau_{1/2}$) of 500 ms for GCaMP6s (2), so that shorter events were excluded. In this way an event trace was generated that contained supra-threshold events with values ≥ 2 and zeros for the epochs where the original fluorescence trace did not reach the detection threshold. To this end, we computed the activity level as the average event trace per behavior. We defined maximally active cells when the activity level for a certain state was greater than 50% of the sum of the average activity levels over all assessed behaviors. For the classification of the feeding-max and the food approach-max neurons we only considered the first five bouts. Note that for unspecific neurons, no behavior dominated the activity profile, and for inactive cells no significant calcium transients were measured. For quantitative analysis, we went back to the original signal and extracted the mean activity during the respective behavior for all neurons from the different functional identities.

As described above in the free-feeding task, we classified the neurons into a certain sleep clusters (**Fig. 3*B*** and ***C***) when the average activity level in a state exceeded 2/3 of the sum of average activity levels in all sleep-wake states. When this latter sum was composed of more than 90 % by two states, the cell was classified as two-state-max (e.g. wake/REM-max). All other fractions were considered unspecific or inactive. For the above described classification only sleep states of 14 s duration or longer were taken into account. For the quantitative comparison we considered the average fluorescence from the non-thresholded trace for each sleep-wake state. We also assessed the effects of raising the threshold for significant events from 2 to 3 or 4 normalized ΔF/F but did not find striking differences (see ***SI Appendix,*** **Fig. S5*G* - *I***).

To confirm the above described classification we performed an unsupervised clustering using affinity propagation (13). As a distance metric we used Pearson's correlation coefficient between the average activity for each behavior of pairs of neurons. This allowed for the extraction of clusters with similar activity profile (functional profiles) rather than finding groups solely according to general high activity. Affinity propagation clustering yielded very similar groups (***SI Appendix,*** **Fig. S5*A* - *F***).

In a synthesis of the classifications we computed a contingency table showing the percentage of cells classified according to their activity profile in sleep and feeding behaviors (**Fig. 4*A***). To better visualize differences and unexpected counts, we calculated the Pearson's standardized residuals, S, for each pair of functional types (***SI Appendix,*** **Fig. S6*A***):

$$S(Y_{i,j}) = \frac{Y_{i,j} - \mu_{i,j}}{\sqrt{\mu_{i,j}}}$$

With:

$$\mu_{i,j} = \frac{\sum_i Y_{i,j} \cdot \sum_j Y_{i,j}}{n}$$

Where $Y_{i,j}$ is the observed count of neurons being classified into a given feeding cluster i and a sleep cluster, j, $\mu_{i,j}$ is the expected count for a given combination and n is the total number of neurons.

We assessed whether the rate of day-by-day re-classification for each functional cluster was different from chance, the null hypothesis being that each cluster is randomly selected from the entire population of neurons (***SI Appendix,*** **Fig. S12*D***). To this end we performed 1000 random draws of the neurons for the different clusters and asked how unlikely the observed re-classification rate after one day was, given the random selections. Note that we selected the same number of neurons per cluster as was seen in the true data on the next day. All comparisons for the cell classification were done with all the recorded neurons, irrespective of animal identity.

Activity tracking over bouts

To track the activity of the cell population or neurons belonging to a functional cluster over feeding and approach, we extracted the average activity for all feeding- and food approach bouts separately (**Fig. 2*E***, ***F*** and ***SI Appendix,*** **Fig. S3*G***, ***H***). The animals showed 17.6 ± 5.5 (mean ± standard deviation) feeding or approach bouts, the minimum was 10. Although we only plotted 10 bouts for the analysis we considered all available data. Linear regression was performed on each animal (***SI Appendix,*** **Fig. S3*G*** and ***H***) or each neuron (**Fig. 2*E*** and ***F***) separately rather than on the average of them. This ensures that the linear relationship is consistently found over the animals and cells and that it cannot be explained by strong trends from single data points. In the case of the approach-max activity over approach bouts visually an exponential decay fit better the observations than a linear one and this was corroborated with a smaller R^2.

We also directly compared the cell activity and classification during the first 200 frames of feeding, food approach or non-feeding behavior (start) with the last 200 frames (end) of the respective behavior (***SI Appendix,*** **Fig. S4)**. We classified the neurons according to the start time stamps and compared the average activity between the different functional clusters during the start or the end frames of the recording (***SI Appendix,*** **Fig. S4*B***). We also compared the fraction of neurons in the different clusters when the cells were independently classified according to the start or the end frames (***SI Appendix,*** **Fig. S4*C***) and assessed how many neurons underwent re-classification (***SI Appendix,*** **Fig. S4*D***). The start portion corresponds to an average of 2.2 feeding bouts and 2.8 food approach bouts, the end is composed of 1.4 feeding bouts and 3.2 food approach bouts.

Activity-similarity correlation

We tested for the association of representation and overall cell activity (**Fig. 4*D*, *E*** and ***SI Appendix,*** **Fig. S6*B***, ***C***). To first obtain a similarity estimate we correlated the activity patterns observed during the sleep-wake states with the average representation of feeding, food approach and non-feeding behavior. Then this similarity value for each frame was in turn correlated to the average normalized ΔF/F from all the neurons during this very frame. A shuffled dataset was generated by randomly permuting the order of the average activity values and correlating them with the pattern similarity 1000 times. The shuffling did not include drawing samples from another sleep or wake state but rather breaking the association with the observed pattern. All correlations were obtained using *corrcoef* in Matlab.

Immunohistochemistry

At the end of the experiments the mice were deeply anesthetized with an intraperitoneal injection of pentobarbital (Streuli Pharma) dosed at 250 mg/kg and transcardially perfused with 5 ml of cold saline (0.9 % NaCl) followed by 20 ml of 4% formaldehyde (Grogg Chemie). Subsequently, the brains were removed and stored in formaldehyde over night at 4 °C for post-fixation. The next day they were transferred into phosphate-buffered saline (PBS) containing 30 % sucrose before freezing. The brains were cut into 40 µm

sections with a cryostat (Hyrax C 25, Zeiss) and collected in PBS with 0.1 % Triton A-100 (Sigma-Aldrich), PBST.

For immunostaining against eYFP or MCH the sections were first incubated for 1 h at room temperature in a blocking solution composed of PBST and 4 % bovine serum albumin (Sigam-Aldrich). Then the tissue was incubated with anti-GFP (1:5000, A0262, Invitrogen) or Anti-pmch (1:500, sc-14502, Santa Cruz Biotechnology) for 24-48 h. After the incubation the brain sections were washed for 5 x 5 minutes and then incubated with an AlexaFluor488 or AlexaFluor555 secondary antibody (1:500 dilution, A96947 for eYFP, A21432 for MCH staining, Invitrogen) at room temperature for 1 h. Cell nuclei were stained with Hoechst (concentration: 1 µg/ml, bisBenzimide H33258, Sigma-Aldrich) mixed into the solution containing the secondary antibodies. The sections were washed again 3 x 10 minutes and then mounted on glass slides. Cover slips were glued on the slides with Fluoromount (F4680, Sigam-Aldrich). Note that the GCaMP6s and ArchT-tdTomato fluorescence was readily visible and no detection was required to determine the expression.

Photomicrographs were taken using an Olympus BX 51 epifluorescence microscope or an Olympus Fluoview FV1000 confocal laser scanning microscope.

For display the image brightness and contrast was moderately adjusted in Adobe Photoshop CC and figures were assembled in Adobe Illustrator CC.

Statistical analysis

All data are displayed as averages with standard error of the mean (S.E.M.). Testing was performed two-sided with a confidence level α of 0.05. Unless stated otherwise the statistical testing included only the graphed data. Test details are only reported for significant results. The data were not systematically tested for normality but were generally tested using parametric tests. When testing for the similarity of patterns the values are bounded between -1 and 1 and so extreme non-normality (heavy tails for example) are unlikely. For the comparison of the activity of groups of individual neurons we observed non-normality. However, we consider that the benefits of including more variance and adjusting for more multiple comparisons directly in a single two-way repeated-measures ANOVA, as compared to multiple Kruskal-Wallis tests, out-weight the contraindication to using parametric statistics on non-normally distributed data. This view is supported by the finding that when comparing the results from two-way RM ANOVA and individual Kruskal-Wallis tests on the datasets from Figure 2*F* and 3*C* we obtain more conservative significance estimates for ANOVA. Hence, for all comparisons of two or more distinct groups in two or more conditions we used two-way ANOVA. For comparisons of pattern similarity including a shuffled dataset (**Fig. 6*E*** and ***SI Appendix,*** **Fig. S6*B***, ***C*** and **S12*A***, ***B***), average population activity (***SI Appendix,*** **Fig. S12*E*** and **S13*E***) or two different time points for functional clusters (***SI Appendix,*** **Fig. S4*B*),** we considered the measures to be repeated by two factors: The shuffling, animal identity or functional cluster, and, the sleep state/behavior, time or intervention factor, respectively. The data in **Fig. 4*E*** and ***SI Appendix*** **Fig. S6*B***, ***C*** were treated as repeated measures by sleep state only. Comparisons between the

different clusters of neurons were treated as repeated measures by one factor only. Except the permutation tests all statistical testing was performed in Prism GraphPad7.

We did not perform power calculations prior to the experiments but the group sizes were comparable to the ones from previous studies (14). The experimenters were not blind to the conditions when acquiring or analyzing the data.

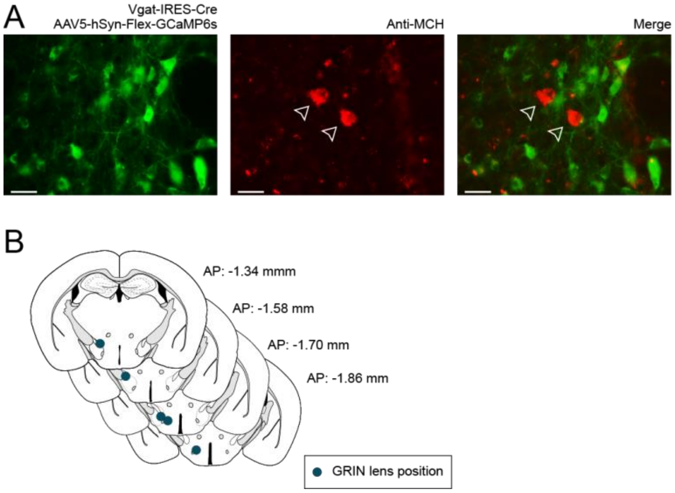

Figure S1 MCH is not co-expressed with LH^{vgat} neurons.

(*A*) Photomicrographs showing GCaMP6s-expressing cells (green, *left*), immunohistochemical detection of MCH (red, *middle*), and merged images *(right*) are shown. Open arrowheads indicate MCH^{+}/GCaMP6s^{-}-expressing neurons. Note that no co-staining between GCaMP6s and anti-MCH was found. Scale bar, 20 μm.

(*B*) Anatomical verification of GRIN lens placement in vgat-IRES-Cre mice.

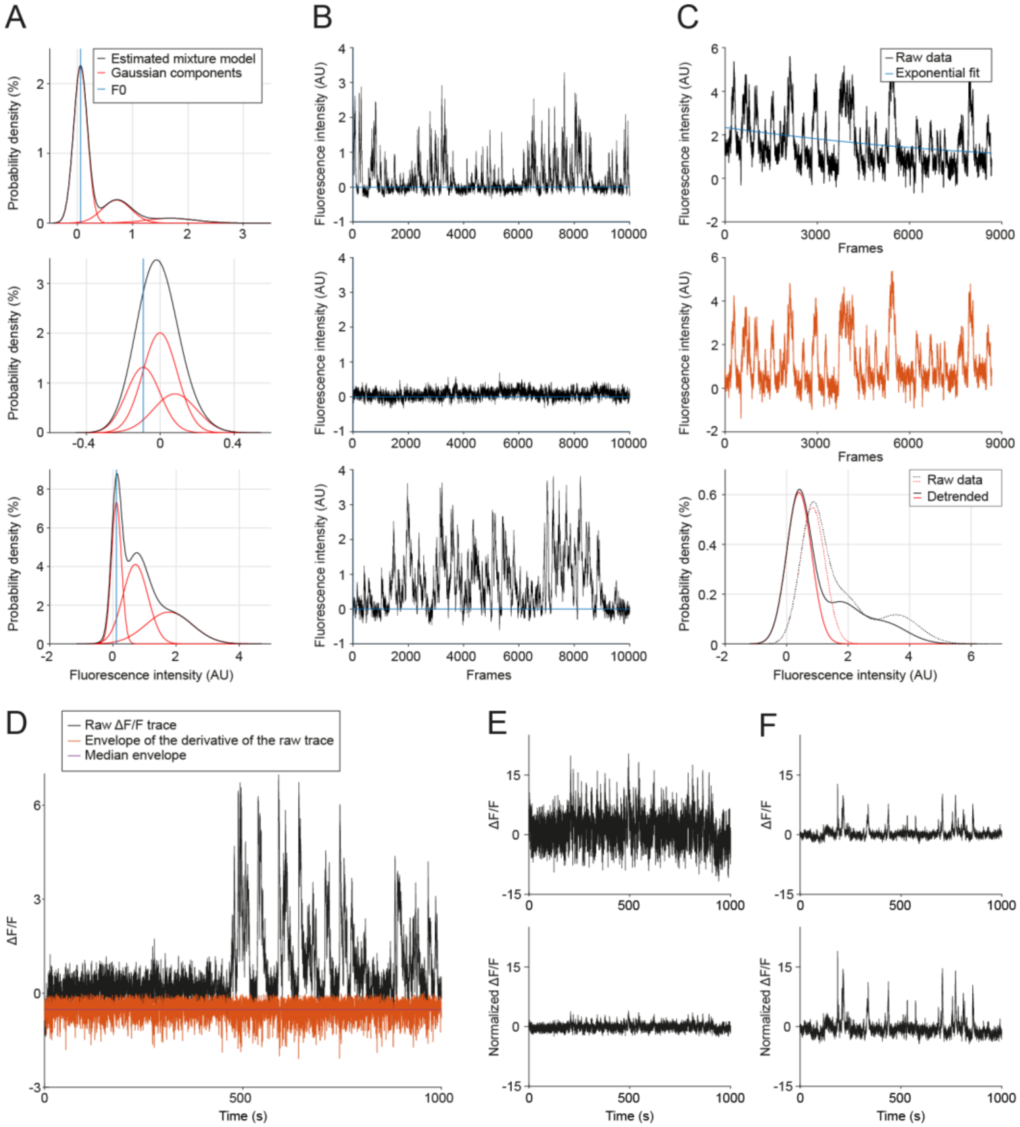

Figure S2 Processing of calcium imaging data

(*A*) Estimation of the baseline fluorescence intensity (F_0) for three detected cells with very different activity profiles is shown. The observed fluorescence intensity distribution from each imaged neuron was approximated by a mix (black) of three Gaussian distributions (red). F_0 (blue) was set to the mean of the Gaussian component comprising, overall, the smallest fluorescence intensity values for three different activity regimes (*top* to *bottom*)

(*B*) Traces after subtracting the corresponding F_0 found in *A*. Note that the clustering performed in *A* yields an accurate estimate of F_0 over a broad range of activity profiles so that the putative periods of low activity or inactivity are centered around zero (blue line).

(*C*) Trends in the raw data (black) are estimated by a first-order exponential (blue, *top*). After de-trending the data (orange, *middle*) the Gaussian mix fitting procedure yields a slightly narrower lowest component that explains more of the data, leading to a more accurate estimation of F_0 (*bottom*).

(*D*) A scaling factor to normalize the traces to their noise level is obtained from the median envelope of the trace's first derivative. The raw trace (black), the positive envelope of the first derivative (orange, here with negative sign for visualization purposes) and its median (purple) are shown.

(*E* and *F*) Example traces from a largely inactive neuron in *E* and a strongly active one in *F* before (*top*) and after (*bottom*) scaling to the 'noise' envelope. Note that the signal fluctuations for presumably inactive periods have similar amplitudes between the cells after the scaling.

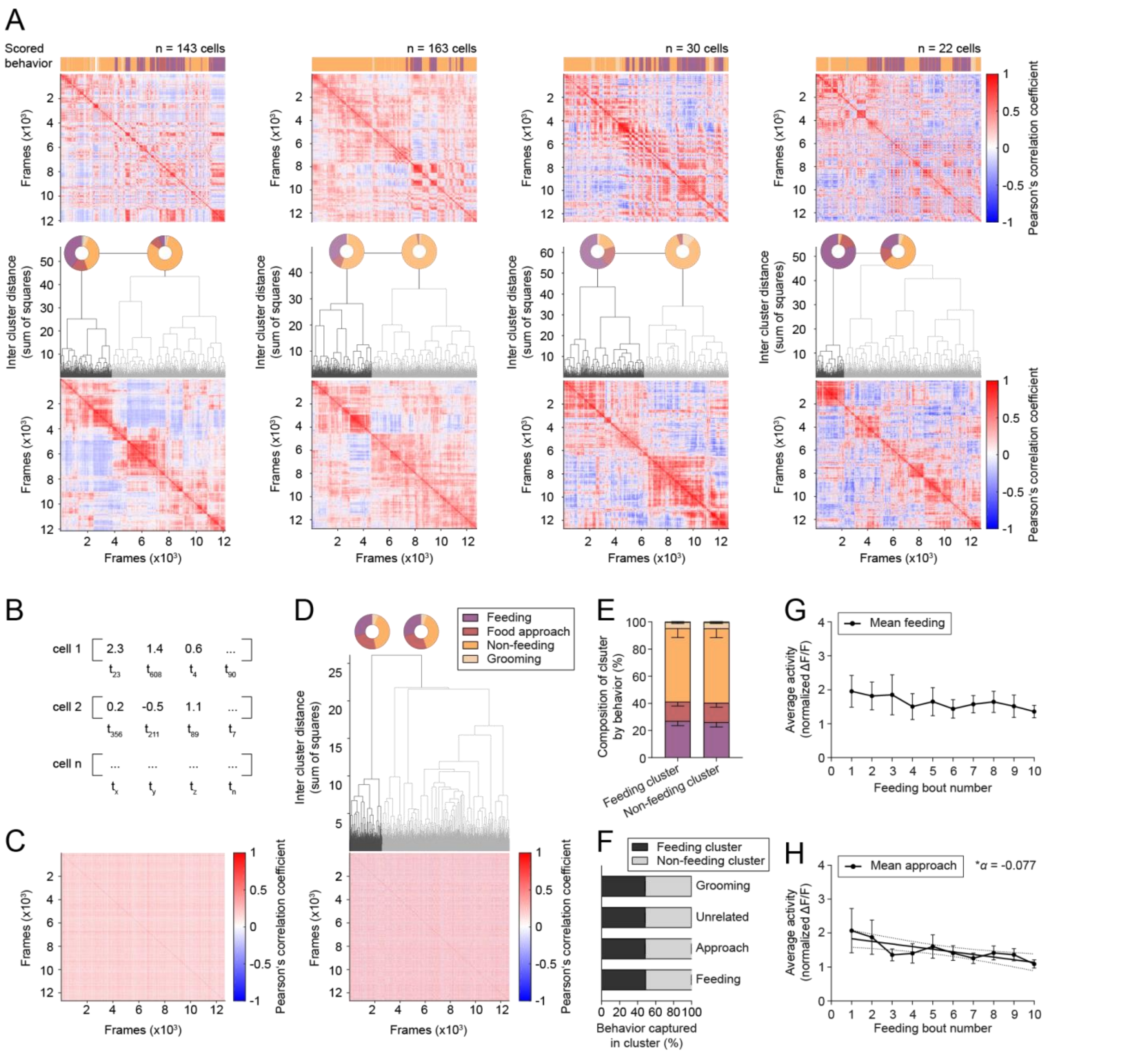

Figure S3 Shuffling the neuronal calcium signals in the time domain abolishes behavioral information.

(*A*) Correlation matrices and hierarchical clustering of the data from the four animals not shown in Figure 1. The scored behaviors are shown on top (colored bar). The obtained dendrogram was cut to yield two clusters that are represented in different shades of gray (*middle*). The pie charts indicate the behavior captured inside the respective cluster. The sorted correlation matrix is shown at the bottom.

(*B*) Schematic illustrating the shuffling procedure. Rather than shuffling the cell identity for each recorded frame, we randomly permuted the frame order for each individual neuron.

(*C*) Example correlation matrix obtained from shuffling the data shown in Figure 1*H*.

(*D*) Hierarchical clustering of the data in *C*. The dendrogram is shown and different shades of gray indicate cluster identities (*top*). Pie charts indicate the cluster composition by behavior. The sorted correlation matrix is shown on the bottom.

(*E*) Bar graph showing cluster specificity by behavior from shuffled data. Sub-divisions are means from 5 animals, for each of which the data were shuffled 1000 times and then averaged. Error bars represent the S.E.M..

(*F*) Cluster sensitivity for the different behaviors is shown for the shuffled data. Sub-divisions and error bars are mean – S.E.M. (n = 5 animals).

(*G* and *H*) Mean ± S.E.M. cell activity over the whole population of LH^{vgat} neurons for individual feeding *G* or approach *H* bouts. Line in *H* represents a significant linear regression ± 95% confidence interval with slope α (n = 5 animals). $^*P < 0.05$.

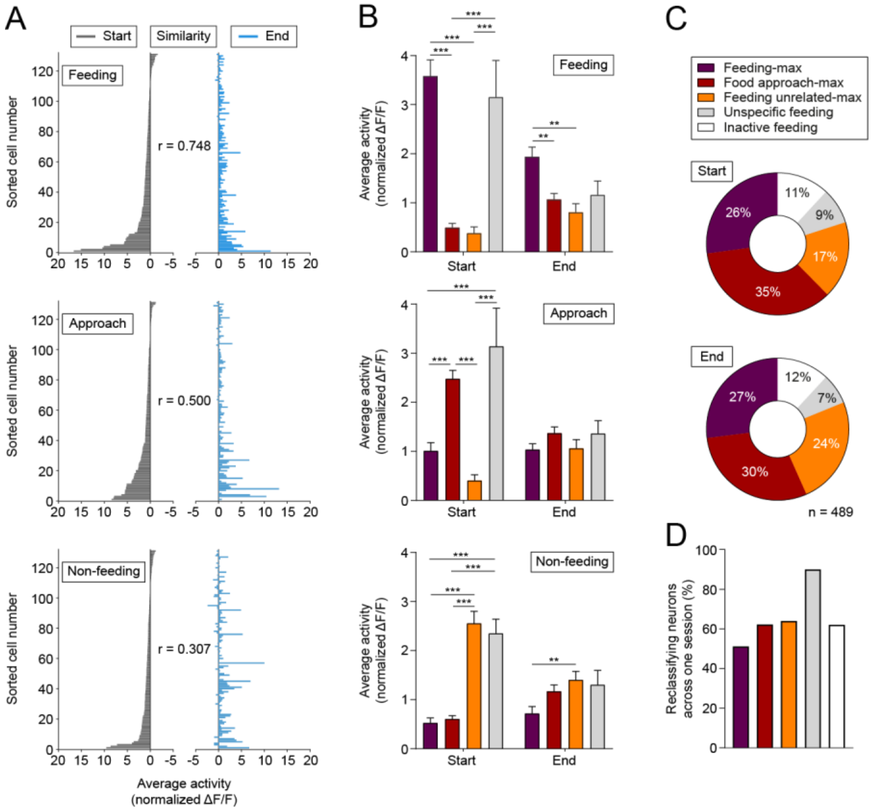

Figure S4 LH^{vgat} neuron activity and reclassification during one behavioral session.

(*A*) Horizontal bar graph showing the average activity for each cell at the start (first 200 frames, *gray*) and the end (last 200 frames, *blue*) of the session for either feeding (*top*), food approach (*middle*) and non-feeding behavior (*bottom*) for a representative animal. Neurons were sorted according to their activity at the start frames. The similarity between the start and the end distributions is indicated by the correlation coefficient, r.

(*B*) Bar graph showing average + S.E.M. activity of LH^{vgat} neurons in each cluster at the start and the end of the behavioral session. The classification was based on the first 200 frames for feeding (top), food approach (middle) and non-feeding (bottom) behavior. Two-way RM ANOVA, $F_{Cluster\ (3,\ 427)} = 36.98$, $F_{time(1,\ 427)} = 18.16$, $F_{Cluster\ x\ time(3,\ 427)} = 23.00$, for feeding behavior, $F_{Cluster\ (3,\ 427)} = 15.98$, $F_{time(1,\ 427)} = 18.45$, $F_{Cluster\ x\ time(3,\ 427)} = 17.74$ for food approaching and $F_{Cluster\ (3,\ 427)} = 29.22$, $F_{time(1,\ 427)} = 10.33$, $F_{Cluster\ x\ time(3,\ 427)} = 17.95$ for non-feeding behavior, with Sidak's *post-hoc* test, $^{**}P < 0.01$, $^{***}P < 0.001$.

(*C*) Pie chart showing the classification of the neurons into functional clusters for the start (*top*) and the end (*bottom*) of the behavioral session (n = 489 cells from 5 animals).

(*D*) Reclassification rate of LH^{vgat} neurons between the start and the end of a behavioral session over all animals, color-coded by functional clusters (n = 489 cells from 5 animals).

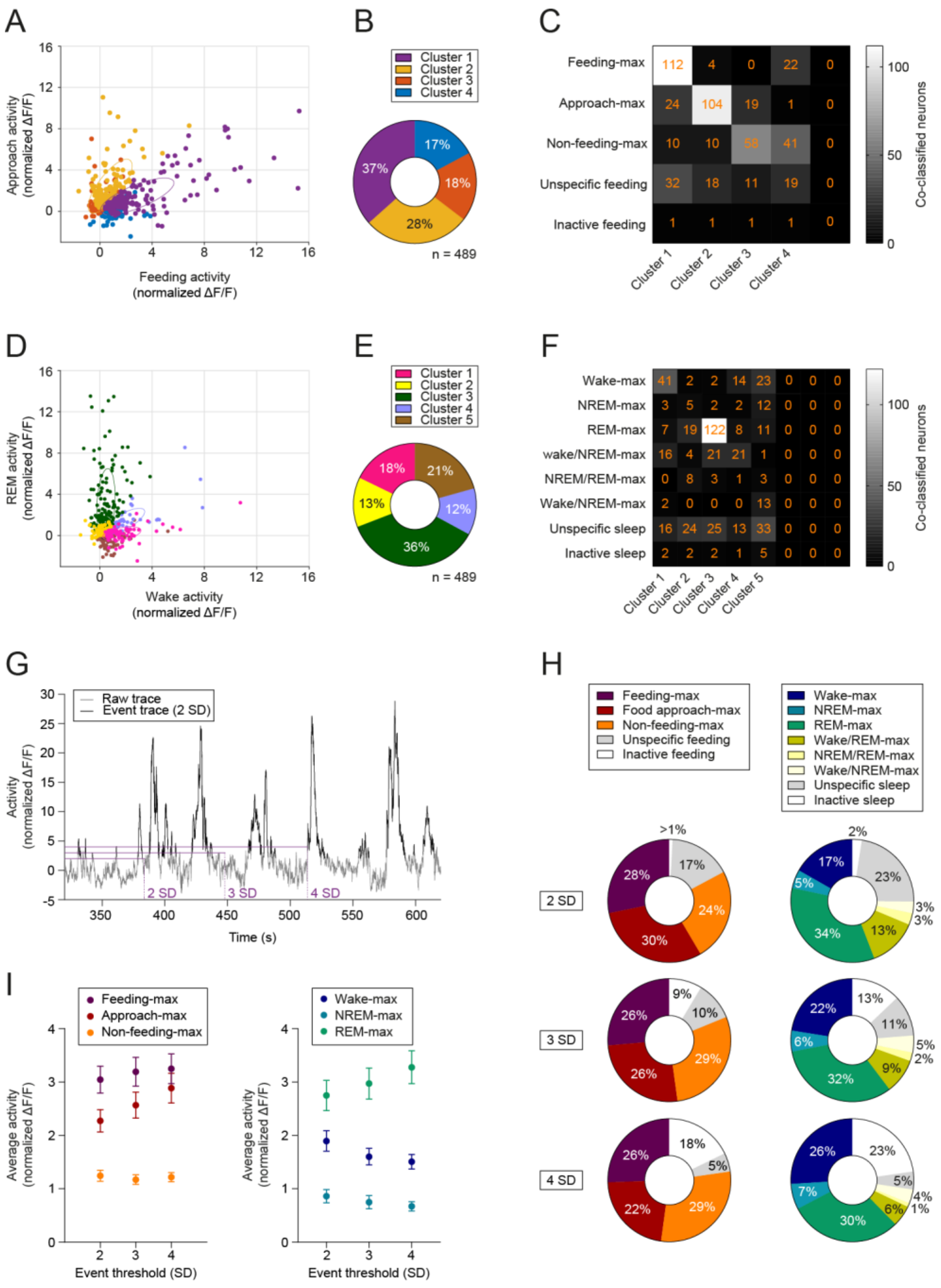

Figure S5 The division into functional cell clusters and their activity is largely unaffected by clustering method or threshold selection.

(*A*) Scatter plot showing the result obtained from clustering the LH^{vgat} neurons according to their mean activity during the first five bouts of feeding or food approach and the non-feeding behavior in the open field using affinity propagation with correlation as the distance metric. The estimated optimal number of clusters from the silhouette value was four.

(*B*) Pie chart showing the proportion of cells in the respective clusters.

(*C*) Confusion matrix to compare the classification with the affinity propagation clustering. Color and numbers represent the number of neurons co-classified with both methods.

(*D*) Same as on *A* but for average activity during sleep-wake states. The estimated optimal number of clusters from the silhouette value was five.

(*E*) Pie chart with the proportion of neurons belonging to the respective clusters.

(*F*) Same as *C* but for the sleep-wake classification.

(*G*) Illustration of event detection for later classification using different thresholds. The raw signal is displayed in gray and the detected events using a threshold of two are shown in black. Different threshold levels are indicated by purple lines (SD, noise standard deviations).

(*H*) Proportion of neurons classified as the different behavioral (*left*) or sleep-wake clusters (*right*) using an event threshold of 2 (*top*), 3 (*middle*) or 4 (*bottom*) noise standard deviations.

(*I*) Average activity the functional clusters when the event threshold was set to 2, 3 or 4 noise standard deviations. Only the activity during the maximally active state is shown for feeding behaviors (*left*) or sleep-wake states (*right*). Dots and bars represent mean ± S.E.M.. Two-way RM ANOVA, $F_{\text{Cluster (2, 1142)}}$ = 64.12 for classification for free-feeding task, $F_{\text{Cluster (3, 780)}}$ = 30.11 for classification for sleep-wake states, with Tukey's *post hoc* test, $P > 0.05$.

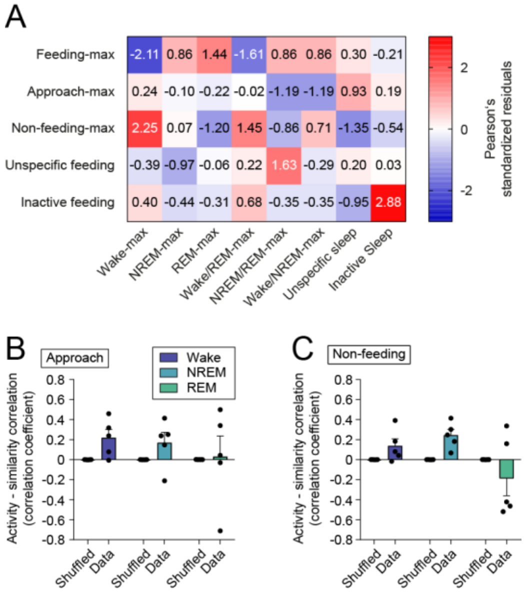

Figure S6 Population activity vectors similar to food approach or feeding unrelated maps are not correlated with elevated calcium transients during sleep.

(*A*) Matrix showing how over- or underrepresented a certain co-classification is as compared to chance (see *SI Appendix*, Methods). Red colors indicate overrepresentation and shades of blue underrepresentation (n = 489 cells from 5 animals).

(*B* and *C*) Bar graphs displaying the average food approach *B* or non-feeding *C* similarity – activity correlation for true and shuffled data (n = 5 animals). Two-way RM ANOVA, $P > 0.05$.

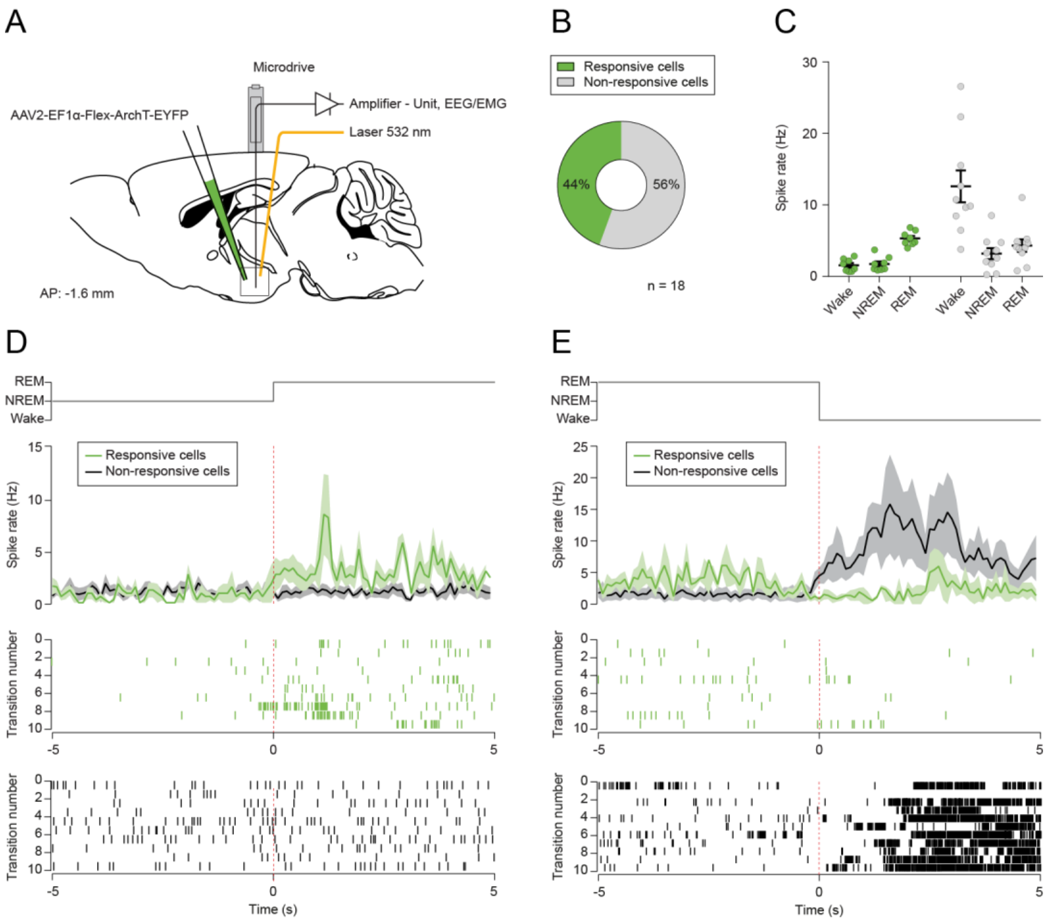

Figure S7 Light responsive LH single units show highest spike rates during REM sleep.

(*A*) Schematic of viral targeting and optogenetic and electrophysiological instrumentation to measure single unit activity in the LH.

(*B*) Pie chart showing the proportions of light-responsive and unresponsive units recorded in the LH (18 units from 3 animals).

(*C*) Spike rates of the recorded units across the different sleep-wake states. Horizontal lines represent mean spike rates and the vertical lines ± S.E.M.. The dots correspond to the individual light-responsive (green) or non-responsive (gray) units.

(*D* and *E*) The average ± S.E.M. spike rate across transitions from NREM- to REM sleep *D* or from REM sleep to wakefulness *E* is depicted for light-responsive (green) and non-responsive units (black). Below the raster plots of one light-responsive and a one non-responsive unit are shown for ten transitions.

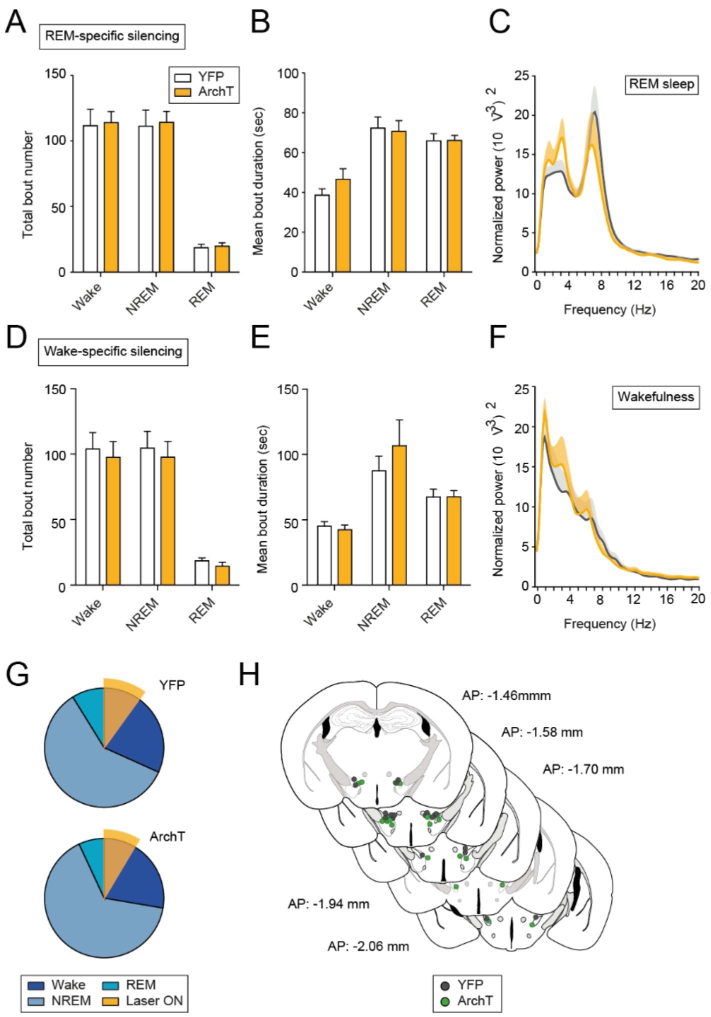

Figure S8 Sleep architecture is not affected by optogenetic inhibition of LH^{vgat} neurons during REM- or wake-specific silencing.

(*A* - *C*) Total bout number of each vigilance state *A*, mean bout duration *B*, or average power spectrum *C*, were not significantly different during the REM sleep-specific silencing experiment in YFP (white, 5 animals) and ArchT (orange, 6 animals) mice. All data are mean + S.E.M., Two-way ANOVA, $P > 0.05$.

(*D* - *F*) Average number of episodes of all vigilance states *D*, bout duration *E*, and power spectrum *F* in YFP control (white) and ArchT (orange) mice. All data are shown as mean + S.E.M., Two-way ANOVA, $P > 0.05$.

(*G*) Average percentage of the vigilance states during the wake-specific silencing experiment for YFP control (*top*) and ArchT (*bottom*) mice. Orange overlay depicts the total time of optical silencing (Laser ON). Two-way RM ANOVA, $P > 0.05$.

(*H*) Anatomical verification of optical fiber placement for YFP (gray) and ArchT (green) mice. AP: anterior-posterior to Bregma.

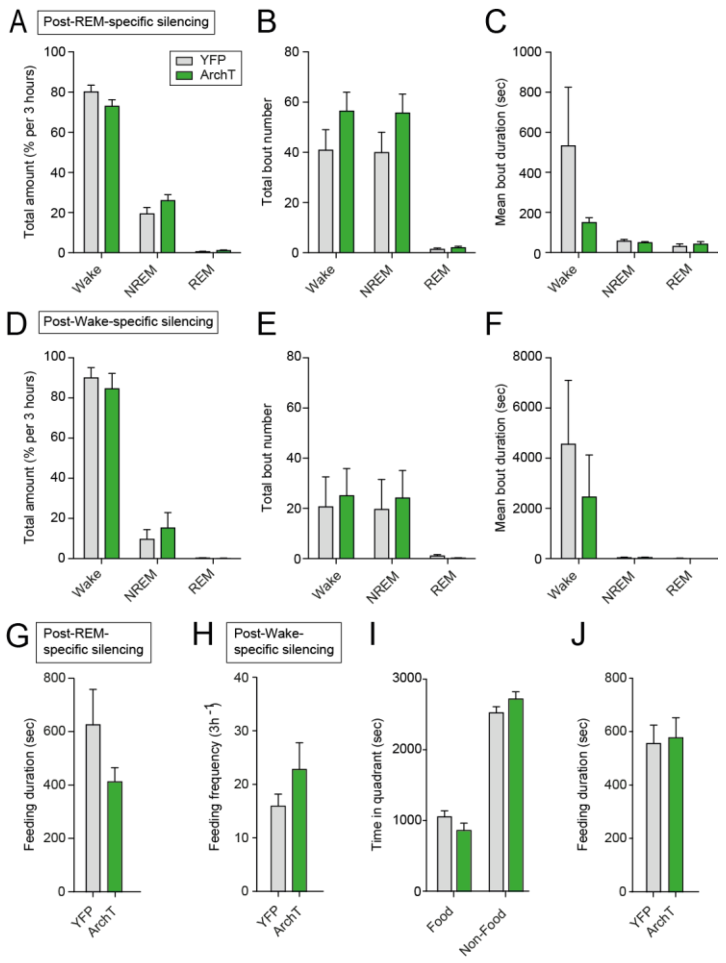

Figure S9 Optogenetic silencing of LH^{vgat} neurons during REM sleep or wakefulness has no effects on sleep architecture at the beginning of the light phase after the stimulation.

(*A* - *C*) Average percentage of each vigilance state *A*, bout number *B*, and bout duration *C* during the three hours following REM-specific optogenetic silencing for YFP control (gray, 5 animals) and ArchT (green, 6 animals) mice. All data are shown as mean + S.E.M., Two-way ANOVA, $P > 0.05$.

(*D* - *F*) Mean percentage of wakefulness, NREM and REM sleep *D*, bout number *E*, and bout duration *F* following the wake-specific optogenetic silencing for YFP control (gray, 5 animals) compared to ArchT (green, 7 animals) mice. All data are shown as mean + S.E.M., $P > 0.05$, Two-way ANOVA.

(*G*) Mean + S.E.M. feeding bout duration for YFP control (gray, 5 animals) and ArchT (green, 7 animals) mice after REM-specific silencing. Mann-Whitney *U*-test, $P > 0.05$.

(*H* - *J*) The feeding frequency *H*, the time spent in the food or the non-food zone *I*, and the feeding bout duration *J* for YFP control (gray, 5 animals) and ArchT (green, 7 animals) mice after wake-specific silencing of LH^{vgat} neurons. All data are shown as mean + S.E.M. Mann-Whitney *U*-test or two-way ANOVA, $P > 0.05$.

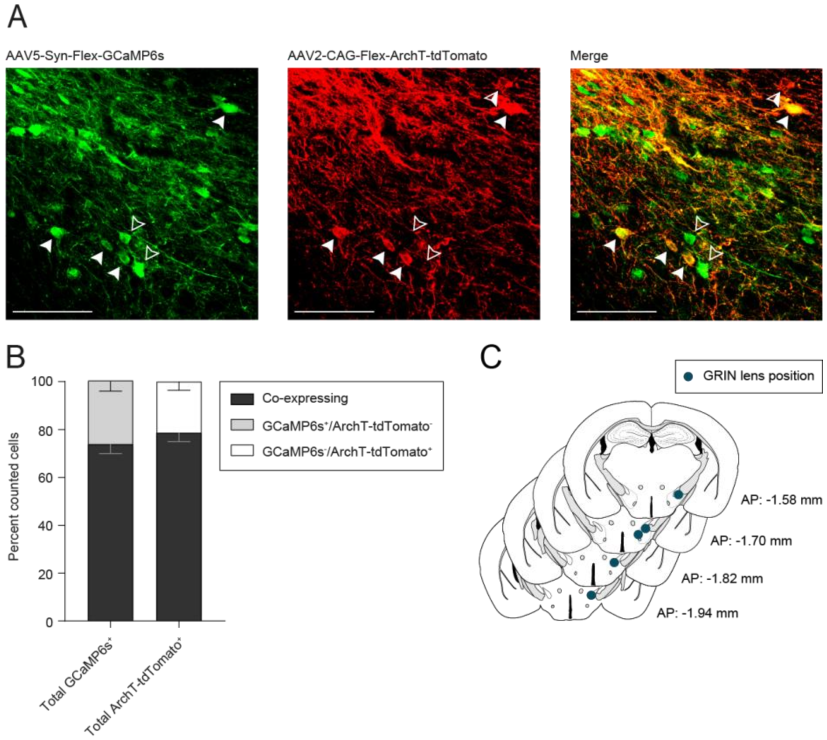

Figure S10 LH^{vgat} neurons co-express GCaMP6s and ArchT-tdTomato.

(*A*) Photomicrographs showing GCaMP6s- (green, *left*) and ArchT-tdTomato-expressing LH neurons (red, *middle*), and the merged images *(right*). Filled arrows indicate double positive neurons, open arrowheads indicate only GCaMP6s-positive and half-filled arrow only ArchT-tdTomato-expressing cells. Scale bar, 100 μm.

(*B*) Quantification of the co-expression GCaMP6s and ArchT-tdTomato. A total of 2609 cells (1613 double-positive, 569 GCaMP6s only and 427 ArchT-tdTomato only) were obtained from four different fields of view in brain sections from five animals.

(*C*) Anatomical verification of GRIN lens placement in the co-transfected vgat-IRES-Cre mice.

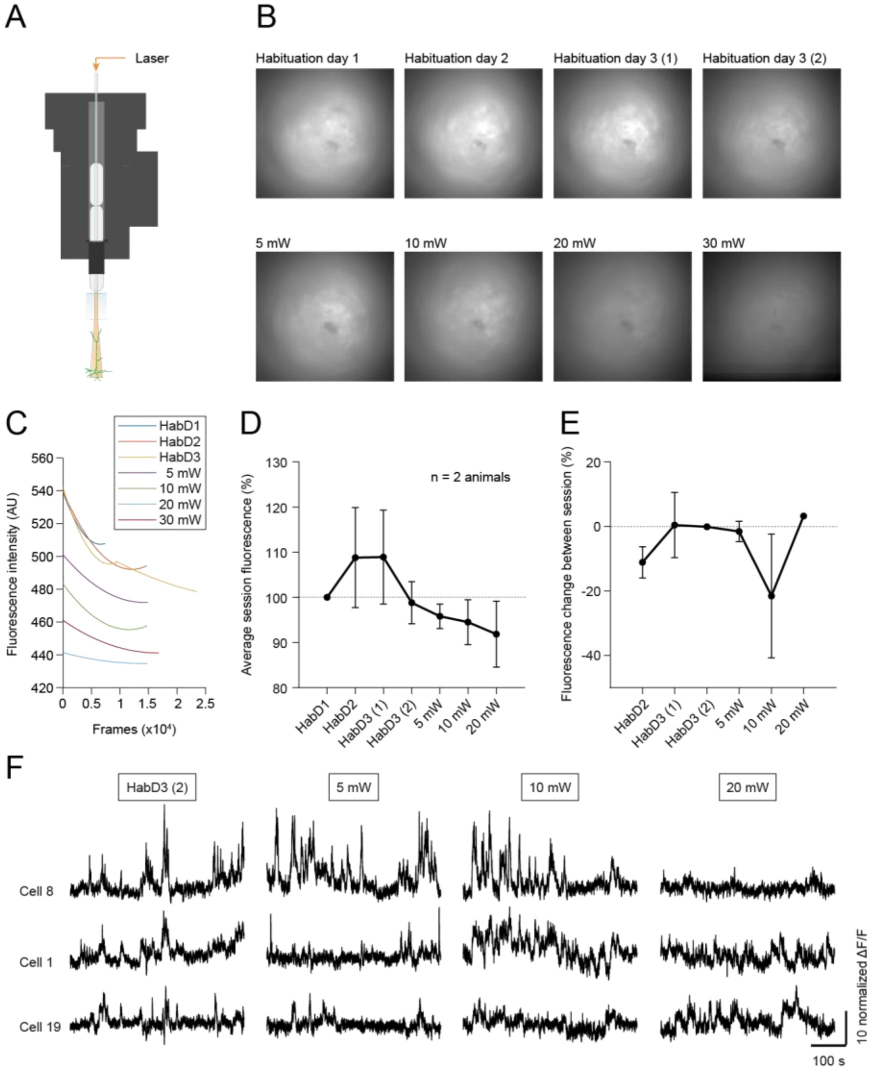

Figure S11 Laser stimulations do not lead to photo-bleaching and do not impair the detection of cellular calcium transients.

(*A*) Schematic of the modified microscope dummy for the delivery of laser light.

(*B*) Average field of view for complete recordings from the LH of an animal expressing GCaMP6s in vgat-positive neurons. The data are obtained after either no intervention (Habituation) or after delivery of a total of 1200 seconds of laser illumination over 4 hours at different powers.

(*C*) Exponential fit of the fluorescence decay within the individual recordings from *B*.

(*D*) Average ± S.E.M. fluorescence for each recording after the respective intervention, normalized to the mean of the first day (n = 2 animals).

(*E*) Average ± S.E.M. decrease of fluorescence intensity after the respective intervention, defined as the jump in fluorescence intensity between the end of the prior session and the start of the next session (n = 2 animals).

(*F*) Representative calcium traces of three example neurons from one animal for different conditions. Note that the calcium transients are unaffected by laser stimulations of up to at least 10 mW/mm^2.

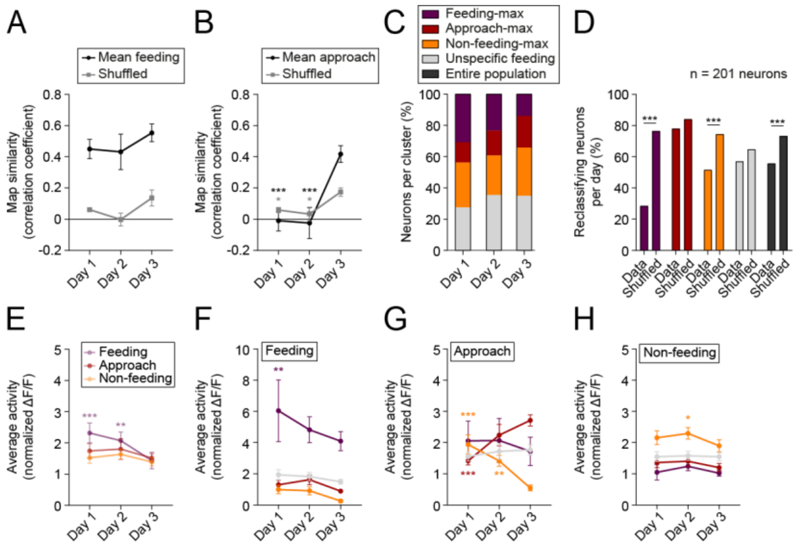

Figure S12 The cellular representation of feeding behavior is stable over multiple days.

(*A* and *B*) Similarity between activity patterns for feeding *A* or food approach *B* from different days as compared to the average pattern from day three. Black color indicates true data, gray are the shuffled observations (n = 5 animals). Dots and error bars represent mean ± S.E.M.. Two-way RM ANOVA, $F_{Shuffle(1, 3)}$ = 75.67 in *A*, $F_{Session(2, 6)}$ = 25.32, $F_{Shuffle\ x\ Session(2, 6)}$ = 25.06 in *B*, with Dunnett's *pos-hoc* test, **P* < 0.05, ****P* < 0.001.

(*C*) Bar graph showing the classification of the neurons into different functional clusters for the different sessions (n = 201 neurons from 5 animals).

(*D*) Daily reclassification rate of the true data over the neurons for all animals (Data) compared to 1000 randomly drawn samples of the same size (Shuffled) color-coded by functional cluster (n = 201 neurons from 5 animals). Permutation test, ****P* < 0.001.

(*E*) Average ± S.E.M. activity of all neurons during feeding, approach and non-feeding behaviors for the different sessions (n = 5 animals). Two-way RM ANOVA, $F_{Behavior\ x\ Session(4, 12)}$ = 3.60, with Dunnett's *post-hoc* test, ***P* < 0.01, ****P* < 0.001.

(*F* - *H*) Average ± S.E.M. activity during the different behaviors by functional classification. Two-way RM ANOVA, $F_{Cluster(3, 197)}$ = 21.71, $F_{Session(2, 394)}$ = 6.36 in *F*, $F_{Cluster\ (3, 197)}$ = 3.52, $F_{Cluster\ x\ Session(6, 394)}$ = 5.76 in *G* and $F_{Cluster\ (3, 197)}$ = 8.9 in *H*, followed by Dunnett's *post-hoc* test against day three, **P* < 0.05, ***P* < 0.01, ****P* < 0.001.

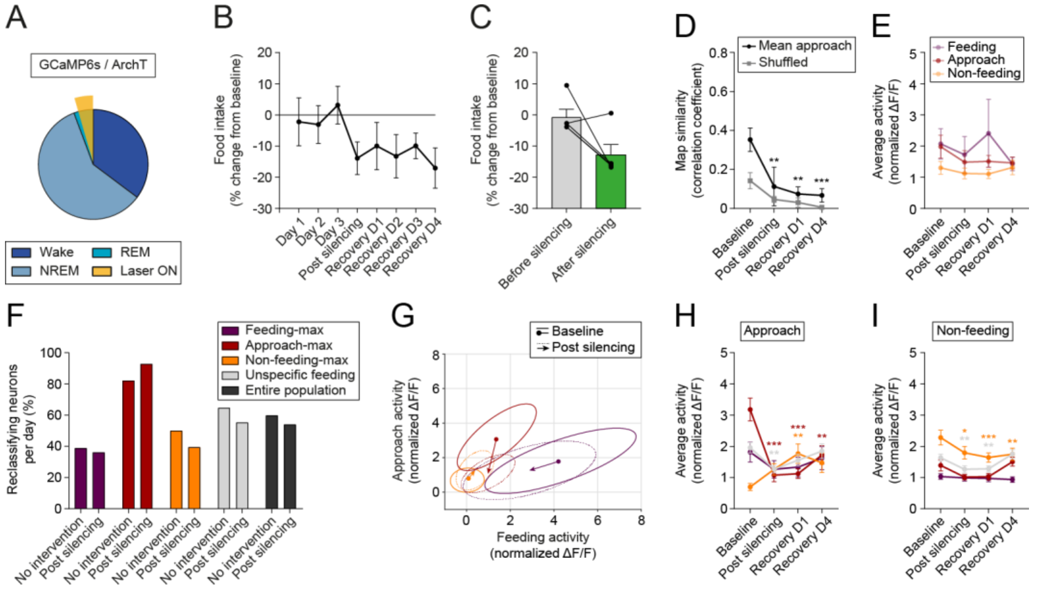

Figure S13 Stimulation efficiency and food intake for imaging group.

(*A*) Average percentage of vigilance states during the REM-specific optogenetic silencing. The orange shading indicates total optogenetic stimulation time during REM sleep.

(*B*) Average ± S.E.M. food-intake change from baseline (mean of day 2 and 3) over the recorded sessions. One-way RM ANOVA, $P > 0.05$.

(*C*) Average ± S.E.M. food-intake change for sessions before (gray) and after (green) optogenetic silencing of LH^{vgat} neurons. Student's paired *t*-Test, $P > 0.05$.

(*D*) True average population vector similarity (black) and shuffle (gray) ± S.E.M. between the approach vectors across experimental timeline (n = 5 animals). Two-way RM ANOVA, $F_{Session(3, 12)} = 7.61$ with Dunnett's *post-hoc* test, $^{**}P < 0.01$, $^{***}P < 0.001$.

(*E*) Average activity ± S.E.M. of all LH^{vgat} neurons for feeding, approach and non-feeding behaviors across the experimental timeline. Two-way RM ANOVA, $P > 0.05$.

(*F*) Daily reclassification rate of LH^{vgat} neurons over all animals with or without intervention, color-coded by functional cluster.

(*G*) Activity (normalized ΔF/F) of feeding-, food approach- and non-feeding-max LH^{vgat} neuron activity before (baseline) and after REM sleep-specific optogenetic silencing (post-silencing) during feeding and food approach behaviors. Dots and arrow heads indicate average activity, solid and dashed ellipses represent the mean-centered covariance.

(*H and I*) Average activity ± S.E.M. of the different functional clusters across the experimental timeline for food approach *H* and non-feeding behavior *I*. Two-way RM ANOVA, $F_{Session(3, 549)} = 6.11$, $F_{Cluster\ x\ Session(9, 549)} = 4.62$ in *H* and $F_{Cluster(3, 183)} = 9.80$, $F_{Session(3, 549)} = 7.92$, $F_{Cluster\ x\ Session(9, 549)} = 2.16$ in *I*, with Dunnett's *post-hoc* test against baseline, *$P < 0.05$, **$P < 0.01$, ***$P < 0.001$.

Movie S1 LH cell activity across feeding behavior and sleep in GCaMP6s-transduced LH^{vgat} animal.

Representative *in vivo* recording of calcium transients from three GCaMP6s-expressing LH^{vgat} neurons (*top left & right*) during free-feeding and sleep recordings. Concomitant to imaging data, mouse video tracking (*bottom left*) and EEG/EMG traces (*bottom right*) were recorded. The position or food intake of the animal (purple or orange, *top right*) and the sleep-state (gray, *bottom right*) are indicated. The video is played at 4 times the original speed.

SI References

1. L. Vong, *et al.*, Leptin Action on GABAergic Neurons Prevents Obesity and Reduces Inhibitory Tone to POMC Neurons. *Neuron* **71**, 142–154 (2011).
2. T.-W. Chen, *et al.*, Ultrasensitive fluorescent proteins for imaging neuronal activity. *Nature* **499**, 295–300 (2013).
3. G. Paxinos, K. B. J. Franklin, *The Mouse Brain in Stereotaxic Coordinates* (Elsevier Academic Press, 2004).
4. X. Han, *et al.*, A high-light sensitivity optical neural silencer: Development and application to optogenetic control of non-human primate cortex. *Front. Syst. Neurosci.* **5**, 1–8 (2011).
5. G. D. Evangelidis and E. Z. Psarakis, Parametric Image Alignment Using Enhanced Correlation Coefficient Maximization. *IEEE Trans. Pattern Anal. Mach. Intell.* **30**, 1858–1865 (2008).
6. P. Zhou, *et al.*, Efficient and accurate extraction of in vivo calcium signals from microendoscopic video data. *Elife*, 1–37 (2018).
7. R. Q. Quiroga, Z. Nadasdy, Y. Ben-Shaul, Unsupervised Spike Detection and Sorting with Wavelets and Superparamagnetic Clustering. *Neural Comput.* **16**, 1661–1687 (2004).
8. S. Jego, *et al.*, Optogenetic identification of a rapid eye movement sleep modulatory circuit in the hypothalamus. *Nat. Neurosci.* **16**, 1637–43 (2013).
9. B. F. Grewe, D. Langer, H. Kasper, B. M. Kampa, F. Helmchen, High-speed in vivo calcium imaging reveals neuronal network activity with near-millisecond precision. *Nat. Methods* **7**, 399–405 (2010).
10. J. T. Vogelstein, *et al.*, Fast nonnegative deconvolution for spike train inference from population calcium imaging. *J. Neurophysiol.* **104**, 3691–3704 (2010).
11. O. K. Hassani, P. Henny, M. G. Lee, B. E. Jones, GABAergic neurons intermingled with orexin and MCH neurons in the lateral hypothalamus discharge maximally during sleep. *Eur. J. Neurosci.* **32**, 448–457 (2010).
12. E. H. Nieh, *et al.*, Decoding Neural Circuits that Control Compulsive Sucrose Seeking. *Cell* **160**, 528–541 (2015).
13. B. J. Frey, D. Dueck, Clustering by passing messages between data points. *Science (80-.).* **315**, 972–976 (2007).
14. J. H. Jennings, *et al.*, Visualizing hypothalamic network dynamics for appetitive and consummatory behaviors. *Cell* **160**, 516–527 (2015).
